# Medicinal Plants of the Family Lamiaceae in Pain Therapy: A Review

**DOI:** 10.1155/2018/7801543

**Published:** 2018-05-08

**Authors:** Cristina M. Uritu, Cosmin T. Mihai, Gabriela-Dumitrita Stanciu, Gianina Dodi, Teodora Alexa-Stratulat, Andrei Luca, Maria-Magdalena Leon-Constantin, Raluca Stefanescu, Veronica Bild, Silvia Melnic, Bogdan I. Tamba

**Affiliations:** ^1^“Grigore T. Popa” University of Medicine and Pharmacy, 700115 Iasi, Romania; ^2^Institute of Chemistry, Academy of Sciences of Moldova, MD-2028 Chisinau, Moldova

## Abstract

Recently, numerous side effects of synthetic drugs have lead to using medicinal plants as a reliable source of new therapy. Pain is a global public health problem with a high impact on life quality and a huge economic implication, becoming one of the most important enemies in modern medicine. The medicinal use of plants as analgesic or antinociceptive drugs in traditional therapy is estimated to be about 80% of the world population. The Lamiaceae family, one of the most important herbal families, incorporates a wide variety of plants with biological and medical applications. In this study, the analgesic activity, possible active compounds of Lamiaceae genus, and also the possible mechanism of actions of these plants are presented. The data highlighted in this review paper provide valuable scientific information for the specific implications of Lamiaceae plants in pain modulation that might be used for isolation of potentially active compounds from some of these medicinal plants in future and formulation of commercial therapeutic agents.

## 1. Introduction

Pain comes in many forms: acute, chronic, visceral, inflammatory, or neuropathic [1, 2]. It is not simply a result of tissue damage but also reflects the influence of many psychological variables such as attention, anxiety, stress [3], suggestion, or previous experiences and may have a significant genetic contribution [4]. Pain accompanies most pathologies present in current medical practice, and 25% percent of Americans, for example, experience pain on a daily basis. Having the numbers on its side, pain became a global public health problem and a leading cause of disability all over the world [5].

As life expectancy is rising and chronical pathologies along with it, the prevalence of accompanying pain is expected to increase yearly, with higher prevalence in elderly patients, where the treatment is also more sensitive [6, 7]. Considering the above, new therapeutic agents with increased efficacy, less side effects, and lower costs and leading to an improved quality of life [8–11] should become one of the primary objectives in modern medical research, together with constant monitoring [12] of the previous mentioned aspects.

The medicinal use of plants as analgesic drugs in folk medicine is an ancient tradition, far older than the current sciences of medicine in developing countries [13, 14]. According to estimations, up to 70,000 plant species are used ethnomedicinally worldwide. Effects of herbal extracts have been studied by different pain tests including writhing test, light tail flick test, tail immersion test, hot-plate test, and formalin test [15].

The exploration for new analgesic combinations from the enormous arrays of medicinal plant resources is growing. This is because such information holds guarantees for the finding of new therapeutic agents capable of inhibiting, decreasing, or relieving pain [16–28]. Plants characterize a vast natural supply of appreciated compounds that might achieve primary importance for the expansion of novel drugs [29]. The survey of the effectiveness of plant-based remedies used in the folk medicine has given great reflections because they are cheap and have reduced side effects.

According to the World Health Organization (WHO), about 80% of the world population still relies mainly on plant-based drugs [30], thus lowering at the same time the impact of self-medication side effects [6]. The data in biomedical literature presenting plants with medicinal capabilities are very similar to the array of publications depicting the modulatory effects certain ones have over pain perception.

The Lamiaceae family, one of the most important herbal families, incorporates a wide variety of plants with biological and medical applications. The most known members of this family are a variety of aromatic spices like thyme, mint, oregano, basil, sage, savory, rosemary, self-heal, hyssop, lemon balm, and some others with more limited use [31].

Our main objective was to perform a review of this literature for the specific implications of Lamiaceae family plants in pain modulation and thus aid the constant search for new potential agents of natural origin with analgesic effects.

## 2. Materials and Methods

**Figure 1:**
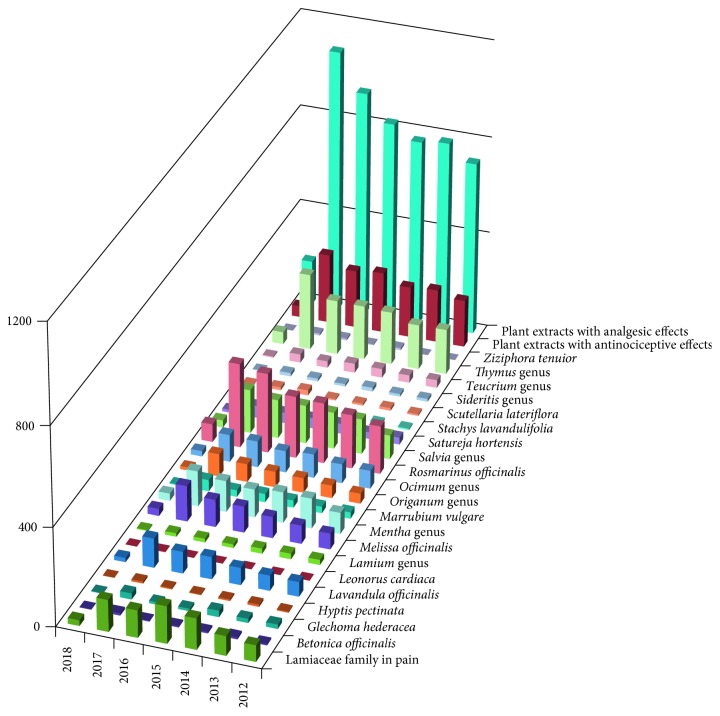
Number of publications according to ScienceDirect.

The search strategy employed in this review includes internationally accepted databases, namely, ScienceDirect, Scopus, Web of Science, and PubMed, using specific keywords of both whole plant products and plant extracts, pain, and analgesic and antinociceptive effects. For investigation, a combination of keywords was used [pain; analgesic; antinociceptive; plant extract] + [*Betonica officinalis*; *Glechoma hederacea*; *Hyptis pectinata*; *Lavandula*; *Leonurus cardiaca*; *Lamium*; *Melissa officinalis*; *Mentha*; *Marrubium vulgare*; *Origanum*; *Ocimum*; *Rosmarinus officinalis*; *Salvia*;*Satureja hortensis*; *Stachys lavandulifolia*; *Scutellaria lateriflora*; *Sideritis*; *Teucrium*; *Thymus*; *Ziziphora tenuior*] + [Lamiaceae; botanical genus]. Case reports, case studies, *in vivo* and *in vitro* relevant studies, and comparative studies were included in this search strategy. Additionally, text books and potentially relevant reviews were explored and included in the reference list. The literature search was confined to the period between 2003 and December 2017. Several articles before 2000 were also included in order to point out the universal interest in natural products with potential applicability in therapy. The dynamic character of the field is reflected in the number of recent publications. For example, a search with the keywords “Lamiaceae family and pain” in ScienceDirect yields 152 titles in 2015, 111 in 2016, and 129 in 2017, and 23 papers will be published in the first months of the next year (Figure 1).

## 3. Species of the Lamiaceae Family with Potential Analgesic/Antinociceptive Effects

### 3.1. *Rosmarinus* Genus

*Rosmarinus* in the Lamiaceae family is a genus of woody, perennial herbs with fragrant evergreen needle-like leaves that is native to the Mediterranean Basin.

#### 3.1.1. *Rosmarinus officinalis*

*Rosmarinus officinalis* L., commonly called rosemary, is a Mediterranean shrubby herb and widely spread in European, American, and Asian countries. It is a common spice used worldwide for culinary, medicinal, and commercial uses, including the fragrance and food industries [32]. The leaves of rosemary (fresh or dries) are used for their characteristic aroma in cooking or consumed in small amounts as herbal tea, while rosemary extracts are regularly used for their natural antioxidant active proprieties to improve the shelf life of perishable foods. Recently, rosemary extracts (E392) have been approved as a safe and effective natural antioxidant for food preservation by the European Union [33].

Phytochemical studies have revealed that leaves contain 0.5% to 2.5% volatile oil. The major components of rosemary oil include monoterpene hydrocarbons (alpha and beta-pinene), camphene, limonene, camphor (10% to 20%), borneol, cineole, linalool, and verbinol. Rosemary contains a widespread variety of volatile and aromatic components. Flavonoids in the plant consist of diosmetin, diosmin, genkwanin, luteolin, hispidulin, and apigenin [34–41]. Additionally, terpenoid components from rosemary consist of the triterpenes oleanolic and ursolic acid and the diterpene carnosol. Phenols in rosemary comprise caffeic, chlorogenic, labiatic, neochlorogenic, and rosmarinic acids. Rosemary covers high amounts of salicylates [42–48].

Modern pharmacological studies have indicated that rosemary and its constituents, especially caffeic acid derivatives such as rosmarinic acid, have various traditional uses in ethnomedicine including analgesic, anti-inflammatory, anticarcinogenic, antirheumatic, spasmolytic, antihepatotoxic, atherosclerotic, carminative, and choleretic applications [44–54], protection against UV and gamma radiation, and amelioration of stress [43].

The powdered leaves are used as an effective natural flea and tick repellent. Activity against certain bacteria including *Staphylcoccus aureus*, *Staphylococcus albus*, *Vibrio cholerae*, *Escherichia coli*, and *Corynebacterium* has been observed. One study found that rosemary oil is most active against “meat spoiling” Gram-negative (*Pseudomonas*) and Gram-positive (*Lactobacillus*) bacteria [49].

Even though rosemary oil is used safely as a food flavoring spice and whole leaves are used as a potherb for seasoning, ingestion of great quantities can be associated with toxicity characterized by stomach and intestinal irritation and kidney damage. While rosemary oil is irritating to rabbit skin, it is not usually considered to be a sensitizer for human skin [55].

Bioactive compounds such as flavonoids, diterpenes, phenols, and triterpenes from plant sources have been traditionally extracted by a conventional solid-liquid extraction. Nevertheless, this extraction technique presents several disadvantages, mainly because it is an arduous, time-consuming process that requires a high consumption of solvents, and in some cases provides low recovery. For that reason, in last years, new promising extraction methods are arising, which introduce some form of additional energy in order to facilitate the transfer of solutes from the sample to solvent in a faster process [54]. Thus, microwave-assisted extraction [56] and/or ultrasound-assisted ethanol, acetone, or *n*-hexane extraction represent alternatives to the conventional method, improving the speed and efficiency of the extraction process and reducing the consumption of solvents [57].

Previous studies have revealed that the rosemary extract may have analgesic and anti-inflammatory effects [58–62]. Therefore, data have shown that the ethanolic extract of rosemary inhibited acetic acid-induced pain in mice with an ED50 of 108.84 mg/kg^−1^ [23]. Furthermore, the extract inhibited the time mice spent licking and shaking induced by formalin injections. Nevertheless, the extract did not display any anti-inflammatory activity as evaluated by uric acid induced-hind limb edema in rats [23]. In an experiment conducted by Emami et al. [34], the effects of *R. officinalis* extract and its major constituent, carnosol, on plasma corticosterone levels and activity of the enzymes cyclooxygenase types 1 and 2 (COX1 and COX2) reduced pain in phase 2 of the formalin test, which was not inhibited by naloxone and/or memantine. In addition, pretreatment of the animals with *R. officinalis* extract and/or carnosol reduced the formalin-induced inflammation. Moreover, the extract and carnosol did not affect plasma corticosterone levels compared with the control group. Interestingly, both the extract and carnosol inhibited COX1 and COX2 activities. Going one step further, one can conclude that *R. officinalis* extract and carnosol suppress pain and inflammation induced by formalin injection, which may be due to inhibition of the activity of COX1 and COX2 enzymes.

### 3.2. *Marrubium* Genus

*Marrubium* is a genus of flowering plants that are included in the Lamiaceae family and are found in the temperate regions of Europe, North Africa, and Asia as far east as the Xinjiang region, and some species are also naturalized as far as North and South America. *Marrubium*, known as horehound or hoarhound, counts approximately 40 species of flowering plants native to the temperate countries of Europe, northern Africa, and Asia.

#### 3.2.1. *Marrubium vulgare*

*Marrubium vulgare* L., commonly named as “marimba” or “marroio” in Brazil and white horehound in Europe, is regularly used in traditional medicine to cure a diversity of maladies [63, 64].

Phytochemical investigations on different parts of *M. vulgare* have reported the presence of alkaloids, lactones, steroids, tannins, a series of phenylpropanoid esters, diterpenoids [65], and flavonoids [64], together with their derivatives. Marrubiin, a furano labdane diterpenoid, was found to be the major chemotaxonomic marker isolated from leaves of the plant and exhibits potent antinociceptive properties and vasorelaxant activity [66–68].

Marrubiin, the main active ingredient of *M. vulgare*, seems to be generated as an artifact from premarrubiin during the extraction procedure when high temperatures are involved in extraction or concentration [69].

The leaves and stems are known to have antiseptic, antispasmodic, antidiabetic, diuretic, strongly expectorant, and tonic roles [70, 71]. The intensive modern research and clinical trials have confirmed several capabilities traditionally described to *M. vulgare*, such as antimicrobial against Gram-positive bacteria, antioxidant, analgesic [66, 67], anti-inflammatory [71], and anti-oedematogenic [72]. Furthermore, extracts of this plant have shown some effects on type II diabetes [73] and, recently, on neurological disorders [74, 75]. One study found that marrubiin has dose-related antinociceptive effects. The antinociceptive properties were observed using different routes of administration (systemic and oral), and the effect was sustained over a long period of time.

The great potencies observed in the writhing test and formalin-influenced pain test propose that marrubiin acts by some peripheral mechanism. In the hot-plate test, marrubiin did not increase the latency period of pain induced by the thermal stimuli. Reducing the lactone ring of marrubiin with the formation of marrubiinic acid and two esterified derivatives has conditioned the successful analgesic effect influencing the number of writhes in mice. Marrubiinic acid exhibited a high analgesic effect that has been long established in other experimental models of pain, suggesting the possibility to use it as a new and useful analgesic agent [67]. Marrubiin does not prove any cytotoxicity against 66 cancer cell lines according to the NIH PubMed website [Marrubiin-Compound Summary (CID 73401)]. *In vivo* experimental studies have documented an LD50 of 370 mg/kg body weight [68], and recent data have highlighted a safety limit up to 100 mg/kg body weight when injected into mice [71].

### 3.3. *Sideritis* Genus

*Sideritis* genus counts more than 150 species of plants that are situated primarily in the Mediterranean area and also in Atlantic regions, North Africa, and even Norway, with apparent differences in composition between the same species corresponding to the geographical place of provenience [76]. The species have been used as flavoring agents, widely as ingredients for tea preparation or with medicinal purposes [77] in some areas being listed as an endangered plant. Although the use in traditional medicine has been extensive in the abovementioned places, *Sideritis* species have reduced usage in western medicine [78], because medical literature are offering data mostly on the *scardica*, *lotsy*, and *stricta* species.

#### 3.3.1. *Sideritis scardica*

*S. scardica* Gris. is also known as “Greek tea” or “mountain tea.” The components of *scardica* have been studied through various methods for their presence as well as medical role in both animal and human studies.

By using chromatographic separations (HPLC) and mass spectrometry, one study found six different flavonoid aglycones: luteolin, apigenin, hypolaetin, 4′-*O*-methylhypolaetin, isoscutellarein, and 4ʹ-*O*-methylisoscutellarein [79], and also other components like sterols, coumarins, flavonoids, essential oil, iridoids, terpenoids, and glycosides [76]. The presence of phenolic antioxidants (catechins) correlating to the antioxidant activity of Greek mountain tea was also established [80].

Gas chromatography with mass spectrometry (GC-MS) analysis demonstrated that the composition of *S. scardica* oil samples, however, varies from region to region. In the oil from Macedonia, for example, *α*-cadinol is predominant as compared to the Bulgarian version of the same plant oil which contains mostly diterpenic compounds and octadecenol. Interestingly, none contained menthol, nerol, or geraniol, which are the major components in the *S. scardica* oil from Yugoslavia [81].

For an overview of the *Sideritis* species in the Balkan area, mountain tea was analyzed by mass spectrometry coupled to high-performance liquid chromatography with diode-array detection. The analysis found that it contains 90% phenylethanoid glycosides and flavonoid acetylglycosides [82]. Turkish *S. scardica* oil has *β*-pinene in abundance as compared to the Greek version which contains *α*-pinene primarily. Both these varieties are mainly rich in monoterpene hydrocarbons unlike the ones from Macedonia and Bulgaria, which are poor in these compounds [83]. Significant differences in components have also been proven between the fresh and dried versions of the plant material [84].

An analysis of urine samples from humans who received oral administration of *S. scardica* showed that the flavonoid metabolites were dominant in urine samples and that hypolaetin and isoscutellarein had the largest number of metabolites (methylhypolaetin and methylisoscutellarein glucuronides) together with apigenin [85].

The pharmacological activity of *S. scardica* is attributed to the high content of flavonoid and phenolic compounds. Studies have demonstrated that plants from the *Sideritis* genus have antioxidant, anti-inflammatory, diuretic, antibacterial, analgesic, and antifungal effects [86]. In experimental ex vivo models, *S. scardica* showed a capacity to inhibit human serotonin transporter (hSERT) greater than in rat models [77]. Accordingly, the *in vivo* test in rats showed that *S. scardica* extract administered orally has been associated with psychostimulant and antidepressive effects, being classified as perhaps a substitute for adaptogens and thus useful for other pathologies correlated with depressive or altered mental status like sleep apnea or increased cardiovascular risks [87–89].

The antibacterial activity seems to be influenced by the method of obtaining the extract: carbon dioxide extraction being superior to hydrodistillation and is attributed partially to diterpenes and fatty acids and their derivates and also to other momentarily unknown elements that might be involved [90] but with a certain degree of effect on different types of pathogens.

The antioxidant activity was widely demonstrated, probably due to the content of catechins but not limited to this and has multiple health benefits and implications in pain treatment proving a possible valuable agent in limiting the use of analgesics, anti-inflammatory, and antipyretic forms of self-medication [6, 91].

*In vivo* models demonstrated the anti-inflammatory effects of *S. scardica* over a model of carrageenan-induced rat paw edema and proved gastroprotective activity over ethanol-induced acute stress ulcer in rats and also a promising cytotoxic activity [92], attributing in part to flavonoid constituents (apigenin and luteolin) that can induce cell-cycle arrest and cellular apoptosis *in vitro* [93]. *In vivo* models demonstrated a preventive capacity of *S. scardica* over A*β*-induced memory impairments in transgenic and nontransgenic mice and proved a possible positive effect in Alzheimer's disease, fully rescuing neuronal loss in transgenic mice, thus being flagged as a possible treatment forimproving memory in healthy adults and in dementia patients [78].

The usage of *S. scardica* in traditional and modern medicine has demonstrated various degrees of effectiveness with promising beneficial health results in a long series of pathologies from prevention of anemia, anxiety disorders, major depression, cardiovascular disease, attention-deficit hyperactivity disorder, mental impairment, or neurodegenerative diseases [77] to rheumatic problems [94], inflammatory pain, gastric ulcer [93], pulmonary pathologies (common cold, lung emphysema, bronchitis, and asthma) [85], and also an effective cytotoxic activity [92, 95]. The anti-inflammatory and edema-reducing capabilities should be considered as the basis for further studies of *S. scardica* implication in pain modulation.

#### 3.3.2. *Sideritis lotsyi*

*Sideritis lotsyi* Pit. contains tetracyclic diterpenes (ent-kaur-16-ene and epicandicandiol 7*β*-monoacetate-18-palmitate), rhoiptelenol, hydrocarbon ent-trachylobane, amyrin, trachinodiol, a rare diterpene 16*β*,18-dihydroxy-ent-atisane, and 5-hydroxy-3,7,4′-trimethoxyflavone, but the content is different between *S. lotsyi* and *S. lotsyi* var. *mascaensis* [96]. *S. lotsyi* var. *mascaensis* extracts were studied in a comprehensive analysis for the antimicrobial activity, toxicity, and anti-inflammatory and analgesic proprieties.

A dose of 2 g/kg body weight *S. lotsyi* extracts administered orally in mice did not show any toxic effects; however, a dose of only 250 mg/kg ethanol extract administered orally has shown analgesic proprieties on the visceral pain produced during the writhing test, and the chloroform fraction demonstrated antinociceptive effect. The same extracts manifested anti-inflammatory effect on the early, histamin-mediated, phase of paw inflammation, but much more significant effects were observed in ear inflammation with topical administration. Contrary to *S. scardica*, no antimicrobial effect was noted [97].

#### 3.3.3. *Sideritis stricta*

*Sideritis stricta* Benth. is listed as an endangered plant and is being used as an aromatic and medicinal plant containing essential oils with antimicrobial, cytotoxic, antiviral, and antioxidant properties [98]. The diterpenes composition was identified as sideroxol, 7-acetyl sideroxol, 7-epicandicandiol, linearol (5), ent-7*α*,15*β*,18-trihydroxy-kaur-16-ene, ent-7*α*-acetyl,15,18-dihydroxy-kaur-16-ene, foliol, sideridiol, siderol, and the recently identified ent-1*β*-hydroxy-7*α*-acetyl-15*β*,16*β*-epoxykaurane [99] together with two flavonoid glycosides and a phenolic fraction by means of spectroscopic evidence [100]. Although phenolic compounds did not manifest anti-inflammatory proprieties, the flavonoid glycosides show both anti-inflammatory and antinociceptive capabilities when combined [100]. Similarly to *S. lotsyi*, the acetone extract of *S. stricta* showed lower antimicrobial activity as compared to gentamicin [99], and no extensive data with the implications of *S. stricta* over pain are published.

### 3.4. *Thymus* Genus

*Thymus* genus, part of the Lamiaceae family, consists of over 350 species of aromatic plants with evergreen leaves. Geographically, these plants extend to Asia, North Africa, and Europe. Although more than one species is cultivated for culinary (cheese and liqueur flavor agent) or ornamental use, the most extensively studied in literature is *Thymus vulgaris*. Used for thousands of years in traditional medicine, the effects of *Thymus* species in medicine is wide, from antimicrobial and anti-inflammatory to possible treatment for dementia or oncological pathologies through apigenin [101].

#### 3.4.1. *Thymus vulgaris*

GC-MS and GC-FID analyses revealed that the main active components in one type of *Thymus vulgaris* L. essential oil are thymol (41.0%), geraniol (26.4%), thujanol (42.2% *cis*-sabinene hydrate and 7.3% *trans*-sabinene hydrate), and linalool (72.5%) [102], and others also contain borneol and carvacrol. The chemotypes of thyme are determined based on oil compositions. Geographical provenience and weather influence the chemotype and composition [103], which was demonstrated by a study comparing essential oils from two regions of France (linalool chemotype with 76.2% linalool and thymol chemotype with 47.1% thymol) and two regions of Serbia (geraniol chemotype with 59.8% geraniol and sabinene hydrate chemotype with 30.8% *cis*-sabinene hydrate) [104].

The terpenoids associated with *T. vulgaris* anesthethic capabilities are thymol (2-isopropyl-5-methylphenol) and eugenol (4-allyl-2-methoxyphenol) [105]; moreover, thymol inhibits synthetisation of vitamin K and is implicated in the inhibition of platelet aggregation [106], resulting in potential anticoagulant activity [107].

In animals, hydroalcoholic extracts of propolis *T. vulgaris* showed promising results in the treatment of dermal leishmaniasis or *Tetranychus urticae* [108, 109]. *T. vulgaris* also has a spasmolytic, antimicrobial, anti-inflammatory, immunomodulatory, and antioxidant capabilities, these effects being attributed to the thymol contained in the volatile thyme oil [110]. Confirming the effect of *T. vulgaris* on respiratory pathologies and the spasmolytic effects underlined in *ex vivo* studies [111], a study also has indicated its promising potential for the treatment of gastrointestinal pathologies in animal models without any toxic potential.

By inhibiting, *in vivo*, TNF-*α*, lipopolysaccharide inflammatory induced cell influx, IL-6, protein concentration in bronchoalveolar lavage fluid, and NF-κB activation in the lung, thymol could be a promising therapeutical agent for acute lung injury [112].

The inhibitory role over the nitric oxide (NO) by limiting iNOS mRNA expression plays a major role in the anti-inflammatory proprieties of *T. vulgaris* extracts [113]. Also, because of the antioxidant capabilities and being an inhibitor of acetylcholinesterase, *T. vulgaris* could be a promising therapeutic agent for neurodegenerative disorders like dementia or Alzheimer's disease [114].

*In vitro* activity of *T. vulgaris* oil confirmed a high antibacterial activity over Gram-positive and also Gram-negative bacteria, though the effect was smaller on the latter [102].

In traditional medicine and in clinical practice, *T. vulgaris* is used, and *T. vulgaris* shows promising results on inflammatory skin disorders [115], scabies, herpes, wounds, alopecia, dental plaque [116], ringworm, and headaches [106]. Moreover, *T. vulgaris* showed a promising insecticidal effect on *Culex pipiens*, the vector for lymphatic filariasis [117], demonstrating an increased importance in many fields.

Probably in part due to the anti-inflammatory and antioxidant capabilities, *Thymus* extracts demonstrated analgesic, anti-inflammatory, and antipyretic activity in mouse models of pain. Therefore, the authors concluded that the extracts of *Thymus* may be used against pain, pyrexia, and inflammation [118], correlating with other similar findings that position *T. vulgaris* as a modulator agent over acute and chronic pain [119]. In clinical practice, comparative effects of *T. vulgaris* and ibuprofen on pain severity associated with primary dysmenorrhea were found [120].

#### 3.4.2. *Thymus pulegioides*

*Thymus pulegioides* L. belongs to the genus *Thymus*, and together with three other species, it has a different phenolic content than *T. vulgaris* [121]. It grows on the European continent, and it is used as an antiseptic in local regions of Portugal [122].

In phytochemical analysis, *Thymus pulegioides* was found to have a high flavonoid content, tannins, and hydroxycinnamic acids. The *T. pulegioides* oil, in one analysiss, was characterized by the presence of high amounts of thymol and carvacrol [122]. The dose-dependent scavenging effect and the chelating activity of *T. pulegioides* are moderate to high, with an increased acetylcholinesterase inhibition [114]. A study in Italy places *T. pulegioides* among the first medicinal plants in traditional medicine and the second most relevant in respiratory pathologies usage [123].

It has an important antioxidant role [124], but as an anti-inflammatory agent, it elicits cell-type-dependent response [125]. Another consideration that increases the medicinal importance of *T. pulegioides* is that it has demonstrated considerable antifungal capacities [122]; however, more data are required to quantify its effect in pain modulation.

### 3.5. *Satureja* Genus

*Satureja* genus consists of aromatic plants of the Lamiaceae family that are related to rosemary and thyme. It is native to the North African region, southern and southeastern European regions, and the Middle East and Central Asian parts of the globe. A few species found on the American continent were formerly included in *Satureja* genus but were thereafter moved to other genera.

#### 3.5.1. *Satureja hortensis*

*Satureja hortensis* L., also known as summer savory (culinary herbs), is an annual aromatic plant with origin in the Mediterranean region and wide distribution in the Mediterranean Sea region, Black Sea, Central and Southern Europe, Asia Minor, and Siberia, but nowadays cultivated worldwide [126]. The floral parts and leaves from the plant are used as aromatic spice. It is also used in medicinal purposes as decoctions, plasters, and compresses.

The main constituents of the plant were carvacrol, *γ*-terpinene, *p*-cymene, *α*-terpinene, and myrcene. The only notable sesquiterpene is *β*-bisabolene [126].

Regarding the biological activity, extracts from *S. hortensis* are covering a large spectrum of pathological conditions [127–132]: antimicrobial activity, antioxidant activity, cytotoxic activity, insecticidal activity, fumigant toxicity, insect repellant activity, antinociceptive and analgesic activity, antileishmanial activity, genotoxic activity, anti-inflammatory activity, effects on immune system, effects on productive performance, acaricidal activity, antidiarrheal activity, relaxant effect (antispasmoidal activity), antigenotoxic activity, antihepatoma activity, contact toxicity and persistence, effect on vitality and healthiness of cereals, molluscicidal activity, larvicidal activity, antihelmintic activity, inhibition on blood platelet adhesion, aggregation and secretion, effect on rhinosinusitis, amyloid beta protein aggregation inhibitory activity, and matrix metalloproteinase inhibitory activity.

Concerning the analgesic activity, *S. hortensis* extracts (hydroalcoholic extract, polyphenolic fraction, and essential oil of the aerial parts of the herb) were evaluated by use of tail flick, formalin, and acetic acid-induced writhing tests in mice. Results showed that, in the light tail flick test, neither the essential oil nor the extracts could exert any significant effect. The hydroalcoholic extract (2000 mg/kg, p.o.) and the essential oil (200 mg/kg, p.o.) inhibited the mice writhing responses caused by acetic acid. In the formalin test, hydroalcoholic extract (500–2000 mg/kg, p.o.), polyphenolic fraction (250–1000 mg/kg, p.o.), and the essential oil (50–200 mg/kg, p.o.) showed analgesic activity, and pretreatment with naloxone (1 mg/kg, i.p.) or caffeine (20 mg/kg, i.p.) failed to reverse this antinociceptive activity. Authors suggested that antinociceptive effect could be due to the involvement of opioid and adenosine receptors in the antinociception mediation [133].

### 3.6. *Stachys* Genus

*Stachys* genus is one of the largest genera in the flowering plant family of Lamiaceae. Estimates of the number of species in the genus are ranging between 300 and about 450.

#### 3.6.1. *Stachys lavandulifolia*

*Stachys lavandulifolia* Vahl., a type of *Stachys*, also known as mountain tea (Chay-e-Kouhi) has been distributed in a variety of climatic conditions including diverse areas of Europe, Asia, Africa, and Australia. The plant is known as Chay-e-kouhi in Persian, whereas in English it is called Betony. Also, its common names include heal-all, self-heal, woundwort, betony, lamb's ears, and hedge nettle [134].

Based on recent studies on this herb, 79 compounds were identified, representing 98.2% of the essential oil, in which the major components were germacrene-D (13.2%), *β*-phellandrene (12.7%), *β*-pinene (10.2%), myrcene (9.4%), *α*-pinene (8.4%), and Z-*β*-ocimene (5.8%). In another study, spathulenol (35.0%) and caryophyllene oxide (25.6%) were the main components of the oil [135]. Another study revealed the existence of *α*-thujone (0.3%–32.3%), Δ-cadinene (11.6%) and 1,4-methano-1H-indene (10.1%) [136].

The aqueous extract obtained from the aerial parts of *S. lavandulifolia* is used in antipyretic, anti-inflammatory, spasmolytic, sedative, and hypnotic treatment [137]. Also, this plant has antibacterial, antioxidant, anxiolytic, analgesic, and wound-healing effects. Decoctions or infusions of *Stachys* are applied as tonics to treat skin or taken internally for stomach disorders [138].

Some other biological activities of *S. lavandulifolia* were signaled, and the main of those being possibility of abortion depending on the dosage in animals, useful in controlling premenstrual syndrome (PMS) and primary dysmenorrhea symptoms, helps in strengthening stomach and preventing gastric ulcers caused by alcohol consumption, and useful in treating *Leishmania major*. Being useful to treat fatigue, nausea, and vomiting associated with primary dysmenorrhea, it could be a potentially effective treatment for dysmenorrhea, particularly because of its antipyretic and spasmolytic effects. As an undesired effect, it gives rise to failure in fetus survival and, consequently, abortion. Its action on insomnia is approved. It is also known for its antidepressive and appetite-stimulating effects [139, 140].

For the evaluation of the analgesic effect, hydroalcoholic, polyphenolic, and boiled extracts of the aerial parts from *S. lavandulifolia* were prepared, and their analgesic effects were studied in mice using formalin, acetic acid-induced writhing, and light tail flick tests. Results showed that all the tested extracts were able to reduce the abdominal constrictions in acetic acid-induced writhing test. These extracts also significantly (*P* < 0.001) suppressed both phases of the formalin test. In the light tail flick test, none of the extracts showed analgesic activity [141].

In another study regarding antinociceptive effects of *S. lavandulifolia* extracts, the implication of essential oil (EOSl) and (−)-*α*-bisabolol (BIS), its main compound, was studied in algogen-induced orofacial nociceptive behavior in mice. Authors have shown that the treatment with EOS1 and BIS has significantly reduced pain in different orofacial pain tests on mice, but BIS proved to be more effective, significantly reducing nociceptive behavior in all tests including both phases of the formalin test [142].

#### 3.6.2. *Stachys officinalis* (Synonym *Betonica officinalis*)

It is commonly known as wood betony, purple betony, woundwort, or Bishop's wort; it is a perennial herb found in dry grassland, meadows, and open woods in most of Europe, western Asia, North and South America, Africa, and tropical regions. For centuries, *Betonica officinalis* herbs (roots and aerial parts) were used in traditional folk medicine for numerous purposes, either internally as tea or externally as compresses or baths. The beneficial properties include anti-inflammatory [143], antibacterial [144], antifungal, antioxidant [145, 146], and hypotensive activity [147]. Important analgesic effects and implications in the treatment of respiratory tract, gastrointestinal tract, nervous and cardiac systems, and skin and gynecological disorders were also observed. Also, a variety of *Betonica* species are used in food industry to improve the taste in preparation of jelly or yogurt, or as seasonings and flavorings [148].

The chemical composition of *Betonica officinalis* includes polyphenols such as tannins, phenolic acids, flavonoids, alkaloids trigonelline, and stachydrine (a pyrrolidine alkaloid), iridoids, diterpenes, phenylethanoid glycosides, fatty acids, betaine, volatile oils, and choline [149, 150]. According to the literature data, phenylethanoid glycosides, triterpenoids, and flavonoids are considered to be the active components responsible for the biological actions of the genus *Stachys*, but the anti-inflammatory or analgesic effects, or components of it, have not been elucidated completely so far.

#### 3.6.3. *Stachys inflata*

A hydroalcoholic extract of *Stachys inflata* Benth., one of the *Stachys* species from Iran, induced antinociception and anti-inflammatory effects in two well-characterized inflammatory models in rats: carrageenan-induced paw edema and formalin-induced paw licking [151]. Intraperitoneal injection of the hydroalcoholic extract of the aerial parts from nonflowering stems of *S. inflata*, 60 min before induction of inflammation, was capable of attenuating both early and delayed phases of carrageenan-induced inflammation with a dose-related inhibition over the dose range of 50–200 mg/kg. Compared to a standard nonsteroidal anti-inflammatory drug, indomethacin, the hydroalcoholic extract of *S. inflata* inhibited the inflammation more effectively than indomethacin. Moreover, all three doses of the extract significantly inhibited the pain associated with the second phase (inflammatory component) of the formalin test, but with no effect against the first phase (0–5 min).

The obtained data suggest that the anti-inflammatory activity of hydroalcoholic extract of *S. inflata* may be related to the inhibition of the release or synthesis of cyclooxygenase products and polymorphonuclear leukocytes accumulation determined by myeloperoxidase activity. The effects of *S. inflata* extracts (200 mg/kg) on inflammation and myeloperoxidase activity were confirmed by histological examination where the extract considerably reduced the morphological injury and neutrophil infiltration in a carrageenan-induced model of local inflammation.

The results presented in this study are taken as the basis for further investigation on the exact mode of action of individual constituents of the extract. Several components quantified in *Stachys* extracts demonstrated *in vivo* anti-inflammatory and antinociceptive activity in carrageenan-induced hind paw edema and *p*-benzoquinone-induced abdominal constriction tests [100].

#### 3.6.4. *Stachys byzantina*

Khanavi et al. [152] proved that acetone and methanol extracts of *S. byzantina* K. Koch, a species of *Stachys*, native to Turkey, Armenia, and Iran, play a significant role in the inhibition of pain and inflammatory processes by using two inflammatory models, namely, formalin test and carrageenan-induced paw edema.

Dried and finely powdered aerial parts were extracted with acetone at room temperature for 2 weeks in order to isolate and identify an acyclic diterpene ester (phytyl nonadecanoate), two normal alkanes (tritriacontane and hentriacontane), one fatty acid (oleic acid), and two sterols (stigmasterol and lawsaritol). Structures were established by conventional methods of analysis and confirmed by ^1^H, ^13^C NMR, and mass spectral analysis. All three doses of acetone/methanol extracts of *Stachys byzantina* (50, 100, and 200 mg/kg), administered by intraperitoneal injections, significantly inhibited the pain associated with the second phase (inflammatory component) of the formalin test, and the effect of the low dose was predominant. Compared to indomethacin (high dose of 5 mg/kg) as a nonsteroidal anti-inflammatory drug, the extracts decreased licking response in the late phase significantly, with the maximum inhibitory response obtained with 50 mg/kg of the extract.

The authors assumed that the analgesic effects of the extracts are probably mediated by interactions with inflammatory mediators (arachidonic acid metabolites), since the antinociceptive activities were observed in late phase (20 min after formalin injection). In the carrageenan-induced paw edema, both extracts revealed dose-related inhibitory effects, in both early and delayed phases, over the dose range 50–200 mg/kg, similar to a high dose of indomethacin (5 mg/kg). The present data demonstrated that the anti-inflammatory activity of acetone and methanol extracts of *S. byzantina* is probably related to the inhibition of the synthesis or release of COX2 products.

### 3.7. *Glechoma* Genus

*Glechoma* genus is composed of flowering plants in the mint family first described in 1753. This genus is distributed in both northern Asia and Europe. In Asia, however, it is most predominantly seen in China, and it is closely related to *Marmoritis*.

#### 3.7.1. *Glechoma hederacea*

*Glechoma hederacea* L., more commonly known as ground ivy, is a perennial herb with creeping stem that can be found throughout Northern Europe and the neighboring regions of Asia. The aerial parts of the plant (consumed as salad or tea) have been used in both Asian and European traditional medicine as a remedy for several digestive, pulmonary, skeletal, and inflammatory conditions [153]. Active components include several polyphenols such as chlorogenic acid, caffeic acid, ferulic acid, rutin, genistin, rosmarinic acid, quercetin, or genistein [153] and triterpenoids such as ursolic acid and oleanoic acid [154, 155]. Additionally, studies report that *G. hederacea* leaves contain polyunsaturated fatty acids [156] and a type of insecticidal lectin called Gleheda [157].

Current preclinical data indicate that *G. hederacea* has several pharmacological effects. As such, hot water extracts of ground ivy have been shown to exhibit antibacterial, anticancer, insecticidal, and platelet-stimulating activity [157, 158]. Currently, there are no studies specifically addressing ground ivy's effect on pain. However, existing data point out that the plant has potent anti-inflammatory effects. An *in vitro* study revealed that incubating activated macrophages with a ground ivy decoction (3 h in boiling distilled water) led to a significant decrease in nitric oxide production. Furthermore, the authors noted that the expression of some inflammatory cytokines such as IL-12p70 and TNFα was significantly decreased [159]. Similarly, Kim et al. demonstrated that several compounds found in *G. hederacea* inhibit NF-κB production [154]. *In vivo*, hot water *G. hederacea* extract was shown to have an anti-inflammatory effect in a rat model of hepatic inflammation: rats that received 0.5 g/kg *G. hederacea* extract daily for four weeks were shown to have significantly lower levels of inflammatory cell infiltration/activation in the liver [153]. Additionally, several inflammatory markers, such as NF-κB, TNF-*α*, IL-1*β*, and IL-6, were decreased in these animals when compared with the control group.

Other possible mechanisms that make ground ivy a potential candidate as coanalgesic include its effects on extracellular calcium (Ca) levels [160] and on oxidation. Purified ethyl acetate extracts of ground ivy showed a strong antioxidant activity when used as a food additive in two different types of food (pork lard and sunflower oil) [161].

There are no reported side effects following *G. hederacea* administration. However, one *in vitro* study showed that *G. hederacea* ethanol extract concentrations exceeding 100 *µ*g/dl are cytotoxic [160], and several studies now focus on the plant's ability to kill different types of cancer cells [162]. Due to its ability to target and kill cancerous cells, those extracts should also be included in preclinical screenings addressing pediatric cancerous cells (e.g., insulinomas being one of the most frequently encountered types of neuroendocrine pancreatic tumors [163]).

### 3.8. *Scutellaria* Genus

*Scutellaria* genus includes over 350 species, many of which have been used in traditional medicine and are documented to have medical proprieties.

#### 3.8.1. *Scutellaria lateriflora*

*Scutellaria lateriflora* L., also known as American skullcap, is a member of *Scutellaria* genus and is native to North America and is best known for its sedative and anxiolytic effects. The plant is still widely used by herbal medicine practitioners for insomnia, nervous anorexia, headaches, depression, panic attacks, and fibromyalgia [164, 165]. Most often, it is prescribed as a tincture, although teas and tablets are also commercially available, with wide variability depending on the manufacturer and species of *Scutellaria* used [166]. Although rare, possible side effects of chronic treatment include drowsiness, mild digestive upset, and vivid dreaming [165].

The first clinical study assessing skullcap's effect on mood was performed on nineteen patients and had positive results [167]. In 2014, Brock et al. published the results of a larger randomized controlled clinical trial designed to assess the effect of a *S. lateriflora* extract on mood in healthy volunteers. Results indicated that global mood was significantly enhanced in individuals who received 350 mg of plant extract for two weeks without negative effects on energy and cognition [164]. Taking into account the fact that anxiety is a well-known enhancer of pain perception [168], *S. lateriflora* extracts could have clinical value as co-analgesics. Additionally, ethanolic and aqueous *S. lateriflora* extracts have been shown to have potent antioxidant effects, reducing ROS and lipid peroxides in tissue homogenates [169], most likely due to the flavonoids it contains.

*S. lateriflora* contains several active compounds such as baicalin (40 mg/g in a 50% EtOH extract), baicalein (33 mg/g in a 95% EtOH), GABA (1.6 mg/g in EtOH and H_2_O extracts), and glutamine (31 mg/g in H_2_O extract) [170]. Other flavonoids found in *S. lateriflora* include wogonin, oroxylin A, genkwanin, hesperetin, quercetin, rutin, naringenin, chrysin, and daidzein [167]. While its anxiolytic effects are probably related to some of the flavonoids that bind to one of the serotonin receptors [171], *S. lateriflora*'s antioxidant activity is most likely due to its content of baicalein and its glucuronide, baicalin.

Baicalein can be extracted from *S. lateriflora* through alkali solution and acid isolation methods; for a high-purity extract (99.35%), hydrolysis of baicalin and column chromatography purification can be used [172]. As an isolated compound, baicalein has shown not only antioxidant activity but also significant anti-inflammatory activity in several *in vitro* and *in vivo* models, which has made it an interesting drug to be screened as an analgesic.

One study used several extracts from a plant of the *Scutellaria* genus and found that baicalein has a significant analgesic effect in the carrageenan-induced rat paw inflammatory model [173]. Similarly, baicalein was found to significantly decrease pain-related behavior and c-fos expression (a surrogate marker for pain intensity) in the spinal dorsal horn of animals exposed to painful stimuli [174]. A combination of baicalin and catechins was assessed in three widely used animal pain models and was found to have analgesic effects in visceral, nociceptive, and inflammatory pain [175].

Baicalin has also shown some efficacy in neuropathic pain: an *in vivo* study on spinal nerve ligation rats showed that tactile allodynia and thermal hyperalgesia were reversed by intrathecal baicalin administration. Additionally, baicalin significantly enhanced the effect of morphine in neuropathic animals, most likely by suppressing histone deacetylase 1 expression in the spinal dorsal horn [176]. The compound was also shown to be effective in cancer-induced bone pain: both intrathecal and oral baicalin administration reduced cytokine expression and inhibited pain-related signals as assessed by behavioral and biochemical tests [177, 178] in an animal model.

This compound most likely exerts its analgesic effects through modulating the inflammatory process. Baicalein's anti-inflammatory activity can partly be explained by its inhibitory effects on lipoxygenases—enzymes that play a key role in leukotriene and lipoxin synthesis, thus initiating the inflammatory response. Deschamps et al. found that baicalein inhibits both human platelet 12-lipoxygenase and human reticulocyte 15-lipoxygenase-1 [179]. Additionally, Hsieh et al. showed that baicalein inhibits IL-1*β* and TNF-*α* through modulation of the NK-κB pathway [180] while other authors found that it inhibits protein expression of inducible nitric oxide synthase [181] and COX2 gene expression [182]. Pretreatment with baicalein increased the concentration of antioxidant enzymes such as SOD, catalase, and GSH in an *in vivo* model of myocardial ischemic injury [183] and protected cells against lipid membrane peroxidation [184]. However, it is very likely that, taking into account the fact that baicalein is effective also in noninflammatory types of pain, it has other analgesic mechanisms as well. One hypothesis states that baicalein binds to the GABA_A_ receptor, which has a modulatory effect on pain because GABA is the main inhibitory neurotransmitter. When directly injected into the central nervous system, baicalein has strong sedative and anxiolytic effects due to GABA binding [185]. Also, a recently published article indicated that through GABA modulation, baicalin could be used in orofacial pain modulation [186]. Another study also suggested that baicalein modulates both intracellular and extracellular calcium levels [187], which may play a role in cell signaling and pain transmission.

### 3.9. *Ocimum* Genus

*Ocimum* genus species are amongst the best-known medicinal plants, with historical reports of their antimicrobial, immunomodulatory, antistress, anti-inflammatory, antiulcer, antidiabetic, hepatoprotective, chemoprotective, antihyperlipidemic, cardioprotective, antioxidant, antitussive, radioprotective, memory enhancing, antiarthritic, antifertility, antihypertensive, anticoagulant, anticataract, anthelmintic, and antinociceptive activity [188]. As such, several members of the genus such as *Ocimum sanctum*, *Ocimum gratissimum*, or *Ocimum micranthum* have played a significant part in different traditional medicines and are currently considered as potential sources for innovative drugs.

#### 3.9.1. *Ocimum sanctum*

*Ocimum sanctum* Linn., also known as tulsi, is an indigenous plant commonly found in India [189]. In Ayurvedic medicine, it is used in the form of a fresh leaf extract or a decoction with hot water to alleviate muscular pain, joint pain, and severe headache [190]. It contains (−)-linalool (30–40%), eugenol (8–30%), and methyl chavicol (15–27%). Minor constituents are (+)-delta-cadinene, 3-carene, *α-*humulene, citral, and (−)-*trans*-caryophyllene [191]. In recent years, the interest for evaluating the potential benefits of *O. sanctum* extracts in several conditions has significantly increased, especially in the anticancer, antimicrobial, and neurobiology fields. A double-blind clinical trial assessed the effects of oral ethanolic extract of *O. sanctum* on healthy volunteers and concluded that the drug has immunomodulatory effects and can be given for a period of four weeks without any significant side effects [192]. Although less numerous, there are some studies that have assessed the effect of *O. sanctum* extracts on different types of pain, most often inflammatory or neuropathic.

*In vitro*, *O. sanctum* leaf extracts exhibited significant anti-inflammatory effects in LPS-stimulated monocytic cells, reducing cytokine production and decreasing TNF-*α* secretion [193]. Different types of dried leaf extracts were also shown to be effective in reducing carrageenan-induced and leukotriene-induced paw edema [194]. More recently, a triple-blind randomized clinical study compared an ethanolic extract of *O. sanctum* with chlorhexidine mouthwash in regards to their effect on dental plaque and gingival inflammation and found that the two are equivalent. Additionally, the *O. sanctum* extract was better tolerated and had no side effects [195].

Regarding its effect on other pain models, there are several studies that have demonstrated that *O. sanctum* extracts alleviate neuropathic pain. The method of preparation was similar in most study designs: dried tulsi leaves were reduced to coarse powder and then extracted with a mixture of methanol and water (3 : 1) [189, 190] in order to obtain an oral preparation. 50 mg/kg b.w. of *O. sanctum* extract attenuated sciatic nerve transection-induced axonal degeneration, reduction of nociceptive threshold, and motor in-coordination [190]. Kaur et al. orally administered 100 mg/kg b.w. or 200 mg/kg b.w. of *O. sanctum* to rats that underwent chronic constriction injury in the sciatic nerve and found that the extract alleviated cold-induced hyperalgesia, mechanical allodynia, and paw-heat hyperalgesia [196]. In another study, a 200 mg/kg b.w. dose of the extract was used, and the authors concluded that it is effective in preventing vincristine-induced neuropathic pain in rats [189]. The same dose of *O. sanctum* extract was administered in rats with surgically induced focal cerebral ischemia/reperfusion injury and was shown to reduce both neurological deficit and oxidative damage [197].

#### 3.9.2. *Ocimum gratissimum*

*Ocimum gratissimum* L. is widely found in several geographical regions in South America and Africa [198, 199] and still used as a medicinal plant with analgesic activity [198]. It contains several proanthocyanidins, which have been shown to exhibit significant antioxidant activity, and tannins, saponins, steroids, alkaloids, terpenoids, flavonoids, phenols, and cardiac glycosides [200]. *O. gratissimum* essential oil was orally administered to mice with chronic constriction injury and effectively alleviated neuropathic pain most likely due to eugenol's antihyperalgesic activity [199]. The same group demonstrated the efficacy of the aforementioned essential oil for increasing paw withdrawal latency in the hot-plate test and for decreasing formalin-induced hind paw inflammation and pain-evoked behaviors [201]. Another team used the essential oil of *O. gratissimum* in a model of visceral pain (the writhing test) and in the formalin test with equally favorable results [202]. Similar analgesic activity was demonstrated by *O. gratissimum* aqueous and hydroalcoholic extracts in two animal pain models: the acetic acid writhing test and the hot-plate test [198], indicating that it is efficient in nociceptive, neuropathic and inflammatory pain.

*trans*-Caryophyllene, a sesquiterpene from *O. gratissimum*, was shown to have dose-dependent analgesic effects in several experimental models of acute and chronic pain such as the formalin test, chronic constriction injury, and the hot-plate test. The authors evaluated the potential mechanisms responsible for the substance's properties and found that the analgesic effect was reversed by several types of antagonists [203], thus indicating the involvement of both the opioid and endocannabinoid system [204].

#### 3.9.3. *Ocimum micranthum*

*Ocimum micranthum* Willd. or *Ocimum campechianum* Mill., more commonly known as Amazonian or Peruvian basil, has similar anti-inflammatory and antianalgesic effects in several animal models of pain, although it has been reported as less effective on the hot-plate test [205]. The difference in efficacy between plants is most likely due to their different compositions that additionally vary according to the geographical area. While some authors believe that the saponins these plants contain are responsible for their effect on pain [189], others have suggested that the volatile oil eugenol is in fact the most potent antioxidant and anti-inflammatory compound [197].

### 3.10. *Lamium* Genus

*Lamium* genus contains almost 40 herbaceous plants, some of which have been used as remedies for various conditions such as trauma, putrescence, paralysis, leucorrhoea, hypertension, or inflammation [206]. The *Lamium* species contain different concentrations of iridoids, flavonoids, phenylpropanoids, benzoxazinoids, and essential oil [207], which vary according to species and geographical area of cultivation. Although widely used in traditional medicine, there are only few studies that investigate the potential analgesic effects of this genus. One study screened several plants of the *Lamium* genus and concluded that *Lamium purpureum* has potent antioxidant effects, being able to rapidly scavenge free radicals in several *in vitro* assays [150].

 Another screening study assessed potential anti-inflammatory and antinociceptive effects of different *Lamium* species and concluded that *Lamium garganicum* L. and *L. purpureum* L. extracts are as effective as indomethacin, a reference anti-inflammatory drug. In this study, all extracts were prepared by methanolic extraction of air-dried and powdered aerial plant parts (25 g plant in 250 mL methanol), which was then concentrated to dryness, suspended in water, partitioned, and lyophilized. The study showed that 200 mg/kg body weight *of L. garganicum* or *L. purpureum* methanolic extracts alleviate inflammatory pain in a model of ear edema and in carrageenan-induced and prostaglandin E2-induced hind paw edema [206].

### 3.11. *Teucrium* Genus

*Teucrium* genus contains several mostly perennial plants commonly referred to as germanders.

#### 3.11.1. *Teucrium polium*

*Teucrium polium* L. is a perennial wild-growing plant, widely spread in several regions such as South-Western Asia, Europe, and North Africa [208], and has been used in traditional medicine for the treatment of inflammations, rheumatism, diabetes, and ulcers. Two major components of the dried leaf plant extract are flavons and flavonoids [209]; the essential oil contains *α*-pinene (25.769%) and myrcene (12.507), and the methanolic extract contains sinapic acid (15.553 mg/g) and eugenol (6.805 mg/g) [210]. A preclinical study showed that intraperitoneal administration of 100 or 200 mg/kg b.w. per day for two weeks reduced pain-related behavior in the diabetic rat formalin test [211]. A larger dose of 500 mg/kg body weight of ethanolic extract of *T. polium* inhibited carrageenan-induced inflammation and reduced granuloma formation [212]. Another study compared the effect of morphine and *T. polium* extract on the tail flick latency and found the two to be comparable in efficacy [213]. Both the total extract and the essential oil of the plant exhibited analgesic effects on the acetic acid-induced writhing test, thus suggesting it might be effective in visceral pain [214]. Subsequently, a triple-blind, randomized, clinical trial was designed in order to assess the plant's effects on dysmenorrhea. Seventy female students were randomly assigned to receive either *T. polium* powder every six hours for the first three days of their menstrual cycle or 250 mg mefenamic acid. Study results indicated that the two are equally effective, thus concluding that *T. polium* is effective in this type of pain [209].

#### 3.11.2. *Teucrium hyrcanicum*

*Teucrium hyrcanicum* L., also known as “Purple Tails” is a plant native to Iran, which has been also shown to exhibit analgesic and anti-inflammatory activities in carrageenan-induced paw edema, acetic acid-induced writhing, tail flick, and formalin pain tests [215]. A recent study used a methanolic extract of dried aerial parts of *T. hycranicum* and observed that the high flavonoid content of the plant has significant antioxidant effects [216].

#### 3.11.3. *Teucrium chamaedrys*

*Teucrium chamaedrys* L., also known as “The wall germander,” is an evergreen subshrub native to the Mediterranean region of Europe and North Africa, and to the Middle East. It has been used in traditional English medicine as part of the Portland Powder for treating rheumatism and gout [217]. A preclinical study identified teucrioside as the main active ingredient of the plant and concluded that it is effective in inhibiting calcineurin, thus potentially playing a role in reducing inflammatory states [218].

### 3.12. *Hyptis* Genus

*Hyptis* genus, also known in Brazil as “sambacaitá” or “canudinho,” is a genus of aromatic plants in the Lamiaceae family [219]. The genus *Hyptis* consists of approximately 400 species distributed from the southern United States to Argentina [220] and exhibits a major morphological diversity in the Brazilian Cerrado [221].

#### 3.12.1. *Hyptis pectinata*

*Hyptis pectinata* L. Poit. is present very common in gardens, and it is frequently used as tea (decoctions or infusions) and mouthwash to treat inflammation due to being considered a natural antiphlogistic. In Brazilian folk medicine, the infusion of the fresh leaves is used to treat inflammations, bacterial infections, pain, gastrointestinal disorders, skin infections, nasal congestion, fever, cramps, inflammation, orofacial painful conditions and wound healing [222], fungal infections, and HIV.

Also, the plant has cytotoxicity and insecticide properties [223]. *H. pectinata* has an important neurogenic and inflammatory orofacial antinociceptive effects, without interference in the motor performance. The mechanism is currently unknown but seems to be related to vanilloid and glutamate receptors. The opioid system seems unlikely to participate in the antinociception caused by the extract [224]. The local application of dental gel based on *H. pectinata* has anti-inflammatory effect and also prevents alveolar bone resorption and weight loss in animals with periodontitis [223]. The healing effect of *H. pectinata* suggests that this plant may have antileishmanial action [219].

The aqueous extract of *H. pectinata* possesses antiedematogenic properties in the carrageenan-induced paw edema model. The association of the aqueous extract of *H. pectinata* leaves at 200 mg/kg with intraoperative laser therapy can stimulate liver regeneration and cause a reduction in liver mitochondrial respiratory function without altering its phosphorylative activity [225].

The antinociceptive effects of *H. pectinata* can be seen in the volatile oil [226]. The major constituents of oil are 1,8-cineole (12.46%), *α*-pinene (20.51%), and *β*-pinene (13.54%). *β*-Pinene may be considered a partial agonist of *μ*-opioid receptors [227]. Franco et al. [228] suggested that the essential oils have both peripheral and central analgesic actions without opioid system influence, although the central activity was more discrete. GC-MS analysis showed that *β*-caryophyllene (40.90%) and caryophyllene oxides (30.05%) were the main compounds present in the oil.

In 2011, Raymundo published the results that *H. pectinata* essential oil shows peripheral and central antinociceptive effects, likely mediated by opioid and cholinergic receptors, and anti-inflammatory activity through the inhibition of nitric oxide and PGE2 production [229]. The involvement of the opioid system in the antinociceptive activity of *H. pectinata* essential oil was evaluated in the hot-plate model by pretreating mice with an opioid antagonist, naloxone. The results suggest that naloxone reversed the antinociceptive activity of the essential oil. The antinociceptive effects were observed in other tests like acetic acid or hot-plate [230].

### 3.13. *Melissa* Genus

*Melissa* genus contains the perennial herbs from the Lamiaceae family, native from Europe and Asia but cultivated and naturalized in many other places.

#### 3.13.1. *Melissa officinalis*

*Melissa officinalis* L., also known as lemon balm, English balm, garden balm, balm mint, common balm, melissa, sweet balm, and heart”s delight, is an aromatic herb from the mint family (Lamiaceae) that includes two subspecies: *Melissa officinalis* L. subsp*. officinalis,* the common cultivated lemon balm, and *Melissa officinalis* L. subsp. *altissima*, naturalized in New Zealand and known as bush balm. The first information about the usage of the plant was found in Greece, 2000 years ago. In 2007, Khare [231] published the results that the plant has antidepressant, antispasmodic, antihistaminic, and antiviral properties and can be used in cases of anxiety, neurosis and nervous excitability, palpitation and headache, and also in hyperthyroidism.

The known major components of lemon balm are hydroxycinnamic acid derivatives, particularly rosmarinic acid, caffeic acids, chlorogenic acid, and metrilic acid [232, 233], tannins [234], flavonoids, including luteolin, luteolin 7-*O*-beta-D-glucopyranoside, apigenin 7-*O*-beta-D-glucopyranoside, and luteolin 3′-*O*-beta-D-glucuronopyranoside [235, 236], monoterpene glycosides [237], sesquiterpenes, including *β*-caryophyllene and germacrene [237], triterpenes [238], and volatile oils, including citronellal, citral a (geranial), citral b (neral), methyl citronellate, ocimene, citronellol, geraniol, nerol, *β*-caryophyllene, *β*-caryophyllene oxide, linalool, and etheric oil [239].

*M. officinalis* exhibit antiviral effects against Newcastle disease virus, Semliki forest virus, influenza virus, myxoviruses, vaccinia [240], and herpes simplex virus types 1 and 2 [241], HIV-1 [242]. The antiviral effects are mediated by tannin and polyphenol constituents, rosmarinic, caffeic, and ferulic acids [240].

*M. officinalis* has antibacterial effects and can be used to treat oropharyngeal diseases produced by anaerobic and facultative aerobic periodontal bacteria like *Porphyromonas gingivalis*, *Prevotella* spp., *Fusobacterium nucleatum*, *Capnocytophaga gingivalis*, *Veillonella parvula*, *Eikenella corrodens*, *Peptostreptococcus micros*, and *Actinomyces odontolyticus* [243].

Englberger suggests that rosmarinic acid has anti-inflammatory effects because it reduces paw edema induced by cobra venom factor in rats and inhibit passive cutaneous anaphylaxis in rats at doses of 1–100 mg/kg by mouth. The same author says that rosmarinic acid has antithrombotic effects because it inhibits the classical pathway convertase and the alternative pathway convertase [244].

*M. officinalis* has antithyroid effects (inhibit the binding of bovine TSH to human thyroid plasma membranes and adenylate cyclase, inhibit the extrathyroidal enzymatic T4-5′-deiodination to both T3-and T4-5′-deiodination) [245], spasmolytic effects (observed only in *in vitro* studies on isolated duodenum of rat) [246], sedative effects (dose-dependent sedation, inducing sleep and potentiating subhypnotic and hypnotic doses of pentobarbital) [246], and cardiovascular effects (significant reduction in the cardiac rate by the stimulation of cardiac muscarinic receptors) [247, 248].

### 3.14. *Origanum* Genus

*Origanum* is a genus of herbaceous perennials and subshrubs in the Lamiaceae family, native to Europe, North Africa, and much of temperate Asia and can be found in open or mountainous environments. A few species also naturalized in North America and other regions. The plants have strongly aromatic leaves and abundant tubular flowers with long-lasting coloured bracts. The genus includes *Origanum vulgare* L. or common marjoram and *Origanum majorana* L. or sweet marjoram, the two species of *Origanum* that can be used with medicinal purposes.

#### 3.14.1. *Origanum vulgare*

*O. vulgare* is an aromatic, woody-based perennial, native to the stony slopes and rocky mountain areas at a wide range of altitudes in the Mediterranean area (Portugal and Andalusia), Europe (including the British Isles), and south and central Asia [249].

The difference between these two plants is almost indistinguishable (taste aside) to the amateur gardener. In technical terms, the difference between marjoram and oregano is based on the shape of the calyx and not the leaves, how hairy they are, or the growth habit.

There are a lot of information about *Origanum*. So, Hippocrates used *O. majorana* as an antiseptic agent. The ancient Greeks consider *Origanum* as a symbol of love, honour, and happiness. Aristotle declares that *Origanum* is an antipoison. The people from old Egypt used *Origanum* to disinfect and preserve food [250].

The major compound of *Origanum* oil is terpinen-4-ol (26%), *cis*-sabinene (13.3%), *o*-cymene (9.3%), g-terpinen (5.8%), *trans*-sabinene (5.7%), *p*-menth-1-en-8-ol (5.1%), b-thujene (4.9%), and *α*-terpinen (3.5%). The extracts obtained by supercritical CO_2_ presented higher concentrations of oxygenated monoterpenes, without significant differences between fractions 1 and 2. A study from Iran shows that the composition of essential oils in *O. vulgare* was dominant in *β*-caryophyllene, germacrene D, and *cis*-sabinene hydrate [251]. Another study from Italy shows that the main components of essential oil in the *O. vulgare* ssp. *vulgare* were *β*-caryophyllene, thymol, terpinen-4-ol, and *p-*cymene [252]. Biochemical compounds of *O. majorana* are the essential oil and tannins. The difference between the essential oil obtained from *O. vulgare* and *O. majorana* is in quantity (0.67% and 1.5%) [253]. The maximum quantity was obtained in the full flowering stage. The major component is germacrene D for *O. vulgare* and terpinen-4-ol for *O. majorana* [254].

In the folk medicine, *Origanum* was used to treat several illnesses such as spasmodic, antimicrobial, digestive, expectorant, and aromatic for the whooping and convulsive coughs [255, 256]. *O. vulgare* (oregano) and *O. majorana* (marjoram) inhibit the growth of the bacteria and fungus (inhibited the growth of *Candida albicans*) [257] and the synthesis of the microbial metabolites [258, 259]. The leaves of *Origanum* were used to cure diabetes, insomnia, catarrh, and asthma [260]. *O. majorana* has stimulatory properties and vasodilatatory activity [261]. By acting also on cardiovascular system and being used as an adjuvant for diabetes control, *Origanum* subsp. could both prevent and treat more complex diseases associative developed as: atrial fibrillation development [262–265].

### 3.15. *Ziziphora* Genus

*Ziziphora* genus is an aromatic herb of the Lamiaceae family, native to Ukraine, Russia, Siberia, Central Asia, Xinjiang, Afghanistan, Irancaner, Turkey, and Middle East. *Ziziphora* species were used as culinary herb in Iran [266].

In traditional medicine, *Ziziphora* is used as infusion, decoction, and maceration for various purposes such as sedative, stomach tonic, heart disorders, common cold, inflammation, carminative, diarrhea, expectorant, coughing, antiseptic, migraine, fever, and depression. Moreover, essential oils are used for treating some diseases such as edema, insomnia, lung abscess, tracheitis, hemorrhoids, and hypertension [267]. The antimicrobial activity of the essential oil of *Salmonella typhi* Vi-positive makes it useful in the treatment of typhoid fever, too. The plant extract can modulate immune response by induction of CD40 expression on DCs and cytokine production and inhibition of T-cell stimulating activity of dendritic cells in high concentration [268].

#### 3.15.1. *Ziziphora tenuior*

*Ziziphora tenuior* L. may possess an antidepressant-like effect, and its effect is similar to fluoxetine [269]. The composition of the ethanolic extract of *Ziziphora tenuior* contains two new flavonoids named as “ziziphorin A and ziziphorin B,” 1-hentetracontanol [270], ursolic acid [271], oleanolic acid (5) [272], *β*-sitosterol-3-O-*β*-glucoside [273], and apigenin [274].

The composition of *Z. tenuior* essential oil may therefore vary with plant genetics, environmental conditions, extraction methods, and geographic origin, including climate, soil, elevation, and topography. The main components of *Z. tenuior*, which are identified by GC/MS analysis of the extracts, are 53.977% of *p*-menth-3-en-8-ol, 38.481% of pulegone, and 1.651% of *p*-menth-3,8-diene. The essential oil also contained smaller percentages of *β*-pinene; 4a*α-*, 7*α-*, and 7a*α*-nepetalactone; *α*-thujene; caryophyllene oxide; limonene; E-caryophyllene; and terpinolene. *p*-Menth-3-en-8-ol and pulegone are the main components of *Z. tenuior*, and they are responsible for the antimicrobial activities of the essential oil [275]. Essential oils of *Z. tenuior* aerial parts were characterized by high levels of oxygenated monoterpenes, especially pulegone [276].

### 3.16. *Salvia* Genus

*Salvia* genus belongs to the subfamily Nepetoideae in the Lamiaceae family. In traditional medicine, salvia is one of the oldest medicinal plants used by humans, and it is considered as a universal panacea, used for its antibacterial, antiviral, antioxidative, antimalarial, anti-inflammatory, antidiabetic, cardiovascular, and antitumor effects.

*Salvia* can be used as infusion, tincture with diuretic, hemostatic, and spasmolytic activities, volatile oils with antiseptic role, and essential oil with antimicrobial effect.

The pharmacological effects of *Salvia* essential oils are based on the presence of more than 100 active compounds, which can be categorized into monoterpene hydrocarbons, oxygenated monoterpenes, sesquiterpene hydrocarbons, diterpenes, nonisoprenoid compounds and oxygenated sesquiterpenes [277, 278]. The most abundant components are 1,8-cineole, camphor, and a wide variety of thujenes [279].

Analysis made by spectrophotometry and HPLC shows that *Salvia officinalis* L. has the highest total content (1.785 g %) expressed in gram equivalent caffeic acid, and the highest value for rosmarinic acid (728.68 mg %). Rosmarinic acid is the major component, and it has adstringent, anti-iflammatory, antibacterial, and antiviral activity [280]. *S. officinalis* is the most valuable species in terms of biologically active principal contents compared to other species studied, followed by *Salvia verticillata* L. and *Salvia glutinosa* L. [281].

### 3.17. *Leonurus* Genus

*Leonurus* genus natively grows in the temperate zone of Asia and Europe and was lately adapted in America and Africa. About 24 species of *Leonurus* have been identified, of which 13 species are spread in China. Plants belonging to *Leonurus* genus are traditionally used for antigynecological disorder in East Asia, and as sedative in Europe. Chemical investigations of the genus enriched the natural products library and also enlarged the pharmacological applications of this traditional herb [282].

#### 3.17.1. *Leonurus cardiaca*

*Leonurus cardiaca* L. is a perennial herb widespread in Europe, throughout the plains and hills, as well as in East Asia to the Himalayas and eastern Siberia, Northern Africa, and North America [283]. The common name of *L. cardiaca* is motherwort, but it is also known as throw-wort, lion's ear, or lion's tail. For centuries, motherwort extract has been used as a medicinal plant to treat cardiac and vascular diseases, especially tachycardia associated with anxiety, tension, and stress, and also for hypertension to reduce the risk of thrombosis to inhibit artery calcification formation [284].

The ethanolic extract has been prepared by adding 96% ethanol over aerial parts of the plants for 24–36 hours. The supernatant was collected and concentrated by vacuum distillation at a temperature of 50°C. The extract was completely dried under sterile conditions using an autoclave at temperatures lower than 50°C.

In the aerial parts of *L. cardiaca*, many compounds were identified: terpene compounds: monoterpenes (iridoids: leonuride, ajugoside, galiridoside, and reptoside) [285], diterpenes (of clerodane, furanolabdane, and labdane types) [286], triterpenes (ursolic acid, oleanolic acids, corosolic acid, euscaphic acid, and ilelatifol D) [287], nitrogen-containing compounds (leonurine, stachydrine, and amine choline), and phenylpropanoids (lavandulifolioside), as well as flavonoids, phenolic acids, volatile oils, sterols (*β*-sitosterol and stigmasterol), and tannins. The phenolic compounds comprise phenylpropanoid glycosides such as lavandulifolioside (arabinoside) [288], phenolic acids such as chlorogenic, rosmarinic, caffeic, *p*-coumaric, *p*-hydroxybenzoic, vanillic, and ferulic acids, and phenolic glycoside [289]. The volatile oils mainly contain sesquiterpenes such as germacrene D, epicedrol, *β*-caryophyllene, *α*-humulene, and spathulenol and monoterpenes such as *α*-pinene and dehydro-1,8 cineole [290, 291]. Of these, ursolic acid proved a stronger anti-inflammatory activity than indomethacin and acetylsalicylic acid, and furanolabdane-type diterpenes inhibited abdominal cramps more effectively than the parallel-given aspirin or acetaminophen.

Pharmacological studies have established that *L. cardiaca* possesses additional antimicrobial [286, 292], antioxidant [289, 293], anti-inflammatory [294, 295], antinociceptive [296], neuroprotective [297], sedative [298], and even anticancer effects [299]. The findings obtained by Rezaee-Asl and coworkers, using the *formalin*, *tail flick*, and *hot-plate* tests, assess that central and peripheral mechanisms are involved in the antinociceptive activity of the motherwort extract. According to the tail flick test of this study, *L. cardiaca* extract only at the maximum dose (500 mg/kg) could alleviate the pain in all times of tail flick test, whereas the lower doses (125 and 250 mg/kg) reduced only late pain. The formalin test showed that the *L. cardiaca* extract at a dose of 500 mg/kg and 250 mg/kg was more effective in the first and second phases, suggesting peripheral and central antinociceptive mechanism. The second phase of the formalin test is related to a peripheral inflammatory process [296].

As a conclusion, the studies concerning the analgesic activity of *L. cardiaca* extract afford a justification for the use of this plant in pain and inflammatory disorders. Further research should be accomplished for the isolation of new phytochemicals and to fully understand the antinociceptive mechanism exhibited by the plant extract.

As undesirable effects, one can mention the potential to increase the risk of bleeding due to its antithrombotic and antiplatelet effects, and the synergistic sedative effect when associated with benzodiazepines, which may result in coma [300].

### 3.18. *Mentha* Genus

*Mentha* is a genus of plants in the Lamiaceae family, with an estimated number of 13 to 18 species, lacking the exact distinction between them [301]. Hybridization between some of the species occurs naturally. The genus has a wide distribution across Europe, Africa, Asia, Australia, and North America. While the *Mentha* species can be found in many environments, most grow best in wet surroundings and moist soils. The mint stems grow 10–120 cm tall and tend to spread uncontrollably over an indeterminate area; hence, they are sometimes considered invasive. The most common and popular mints for commercial cultivation are *Mentha piperita*, *Mentha spicata*, *Mentha gracilis*, *Mentha arvensis*, and *Mentha suaveolens*. Mint was originally used as a medicinal herb to relieve stomachache and chest pains [302].

#### 3.18.1. *Mentha piperita*

*Mentha piperita* L. (peppermint) is a hybrid of *M. spicata* and *M. aquatica*. This plant was cultivated since the time of ancient Egyptians and established in the Icelandic Pharmacopoeia of the thirteenth century. The list of benefits and uses of peppermint as a folk remedy or alternative medical therapy include biliary maladies, dyspepsia, enteritis, flatulence, gastritis, intestinal colic, and spasms of the bile duct, gallbladder, or gastrointestinal (GI) tract [303].

The phytochemical occurrence in peppermint leaves and oil depends on plant maturity, variety, geographical origin, and processing methods' conditions [304–307]. As fatty acids, there have been found palmitic, linoleic, and linolenic acids [308]. The main components identified in the volatile oil of peppermint are menthol (33–60%), menthone (15–32%), isomenthone (2–8%), 1,8-cineole (eucalyptol) (5–13%), menthyl acetate (2–11%), menthofuran (1–10%), limonene (1–7%), *β*-myrcene (0.1–1.7%), *β*-caryophyllene (2–4%), pulegone (0.5–1.6%), and carvone (1%) [304, 309]. The fresh leaves contain 1.2–3.9% (v/w) of essential oil, while the dried leaves is reported to contain only 21% of the original oil [310].

Carotenoids, chlorophylls, *α*- and *γ*-tocopherols, and ascorbic acid have also been reported in the plant extract [311]. The major minerals in dried peppermint leaves include K, Ca, Mg, and Na, along with smaller amounts of Fe, Mn, Zn, and Cu and trace amounts of Cr, I, and Se [312]. The polyphenols isolated from peppermint leaves include mainly eriocitrin and rosmarinic acid, luteolin 7-*O*-rutinoside, and hesperidin [313, 314].

The extraction of essential oils has been approached through different techniques, of which hydrodistillation is still the most common to achieve volatile oils from medicinal plants, including *Mentha* [315]. In order to diminish the extraction time and for higher extraction yields along with an increased quality extracts, a number of extraction procedures have also been implemented, such as microwave-assisted extraction, pressurized solvent extraction, supercritical fluid extraction, and ultrasound-assisted extraction [316–319].

*In vitro* and *in vivo* pharmacological studies have proved multiple therapeutic effects, which are mentioned as follows: antioxidant capacity (radical scavenging capacity being higher than that of *M. aquatica* or *M. longifolia*) [46, 320], antitumor activity on different cell lines [321, 322], antiallergenic activity [323, 324], antiviral activity with significant results on herpes simplex viruses (HSV-1 and HSV-2) and against human immunodeficiency virus-1 (HIV-1) [242, 325–327], antibacterial activity against different bacterial strains, including Gram-positive cocci and rods and Gram-negative rods (e.g., *S. aureus*, *Salmonella enteritidis*, *Shigella sonnei*, some strains of *E. coli*, *Heli cobacter pylori*, *Haemophilus influenzae*, *Streptococcus pneumoniae*, *Streptococcus pyogenes*, and many other pathogens) [328–331], modulatory effects on hepatic and renal functions [332–334], nervous system actions as analgesic and local anesthetic, and anti-inflammatory actions [335, 336].

The antinociceptive activity of *M. piperita* aqueous extract has been investigated by Yousef A. Taher using *in vivo* tests on mice [336]. According to these studies, the plant extract showed inhibition of acetic acid-induced abdominal constrictions in mice at both 200 and 400 mg/kg doses. The hot-plate test has shown that administration of *M. piperita* aqueous extract (using the same abovementioned doses) caused a significant latency of the response to thermal stimulation. The carrageenan-induced paw edema test disclosed an increase in paw thickness; hence, it is assumed that the aqueous extract has a noninflammatory pain reliever activity, in contrast with previous research when the phytochemical compounds were obtained by the ethanolic extraction [335]. On the other hand, the methanolic extract of different *Mentha* species displayed different analgesic effects, indicating that these effects are species- and extract-form dependent [337, 338]. These findings indicate that the phytochemicals present in the *M. piperita* extract exhibit analgesic effect arising from both CNS and peripheral actions since the response appears to both thermal and chemical pain stimuli. A similar efficacy is characteristic of central analgesics, such as morphine, which inhibits equally inflammatory and noninflammatory pains. The results concerning analgesic effects produced by *M. piperita* strongly recommend this plant as pain reducing and encourage further studies for a better understanding of the nociception mechanism in order to find new options in pain therapy, with less side effects.

Toxicology studies of peppermint oil and its components completed in animals have shown no adverse effects or histopathological modifications. There are no chronic toxicity studies of peppermint in humans, although the use of peppermint oil has been reported as contraindicated in patients with bile duct, gallbladder, and liver disorders. The use of peppermint oil capsules in patients with GI reflux, hiatal hernia, or kidney stones needs also caution [339].

#### 3.18.2. *Mentha spicata*

*Mentha spicata* L., also known as spearmint, originated in Bangladesh and is traditionally used as herbal remedy for various disorders. Hajjaj Yousuf et al. have performed a study which aimed at evaluating the analgesic, anti-inflammatory, and antipyretic effects of *M. spicata* on animal models, using hot-plate, acetic acid-induced writhing test, carrageenan-induced rat paw edema, and yeast-induced pyrexia methods [340]. The hot-plate results suggest a centrally antinociceptive action with a higher pain inhibition at 180 minutes after administration, being comparable to a standard drug. The acetic acid-induced writhing method evaluates the peripherally analgesic action, which took place through inhibition of local peritoneal receptors, most probably by inhibition of cyclooxygenase activity. The anti-inflammatory effect was maintained at a significant level for a 6-hour period, showing efficiency in the late phase of inflammation due to the presence of certain components that interfere with the release of prostaglandins.

Many other research studies on *Mentha* species such *as M. longifolia* [341], *M. arvensis* [342], or *M. villosa* [343] were also carried out regarding the analgesic activity. Although the phytochemical occurrence is not identical, different mechanisms have been consequently involved in achieving the antinociception, with competitive results.

### 3.19. *Lavandula* Genus

*Lavandula* genus includes more than 39 known species, mostly distributed in Arabia, Mediterranean Coasts, Asia, Middle East, and Northern Africa. *Lavandula officinalis, Lavandula angustifolia*, *Lavandula hybrida*, and *Lavandula vera* have been considered as antidepressive, antispasmodic, antiflatulent, antiemetic, diuretic, anticonvulsant, antibacterial, antiepileptogenic, antioxidant, antibacterial, antifungal, sedative, antinociceptive, and gastroprotective effects [344–348]. Lavender comprised over 100 constituents, among which the primary components are polyphenols, anthocyanins, carotenoids, linalool and linalyl acetate, *α*-pinene, limonene, 1,8-cineole, *cis*- and *trans*-ocimene, 3-octanone, camphor, caryophyllene, terpinen-4-ol, and flavonoids [349, 350].

#### 3.19.1. *Lavandula angustifolia*

*Lavandula angustifolia* Mill. is one of the most famous aromatic and medicinal plants [351] used in fresh state or dry condition, containing 1–6% volatile oils (monoterpenic compounds, alcohols, and esters), triterpenic acids, coumarins, flavones, resins, and polyphenols [352]. As medicinal activity, *L. angustifolia* extracts or essential oils possess antispastic, carminative, analgesic, sedative, hypotensive, antiseptic, antimicrobial, antifungic, diuretic, and general tonic action, but little information on lavender analgesic properties is available in the literature.

#### 3.19.2. *Lavandula officinalis*

*Lavandula officinalis* Chaix is used in traditional and herbal medicine for the treatment of pain and in the reduction of the inflammatory pain. In pharmacological and biological tests, extracts, fractions, and essential oils of *L. officinalis* are reported to have analgesic effects. The literature data show that *L. officinalis* extract contains linalool, acetate linalool, monotril, sesquiterpene, luteolin, ursolic acid, coumarin, and umbelliferone. Hajhashemi and Ghannadi [349] showed that the aquatic, alcoholic, and phenolic extracts have antinociception effects in the second phase of the formalin test, but only the phenolic and alcoholic extracts had been able to prevent the first phase of the formalin test. Barocelli et al. (2004) [353] proved that *L. officinalis* leaves inhalation attenuates pain evoked by hot-plate test, and stomach graze induced by high-dose administration of ethanol and ascetic acid. Husseini et al. (2015) [354] demonstrated that *L. officinalis* hydroalcoholic extracts inhibit inflammation and pain induced by formalin and cyclooxygenase (COX) type 1 and 2 activity in mice, using the formalin and hot-plate tests. The administration of the extract (100, 200, 250, 300, 400, and 800 mg/kg, i.p.) has inhibitory effects on inflammation induced by formalin injection into the animals hind paw, effects equal to morphine, dexamethasone, and indomethacin. The extract in 100, 200, and 300 mg/kg significantly reduced heat-induced pain and COX activity in dose-dependent manner.

#### 3.19.3. *Lavandula hybrida*

In 2004, Barocelli et al. [353] demonstrated the antinociceptive and the gastroprotective effects of orally administered (100 mg/kg) or inhaled *Lavandula hybrida* Reverchon “Grosso” essential oil, and its principal constituents linalool and linalyl acetate in rodents. In the hot-plate test, the analgesic activity was observed after oil inhalation was inhibited by naloxone, atropine, and mecamylamine pretreatment, suggesting the involvement of opioidergic as well as cholinergic pathways. Therefore, the lavender oil reveals an interesting analgesic activity mainly relevant after inhalation, at doses devoid of sedative side effect, suggesting the interest for potential application of this oil in aromatherapy.

## 4. Identification of Secondary Metabolites

The identification of secondary metabolites from essential oils was achieved by gas chromatography coupled with mass spectrometry and from aqueous or alcoholic extracts by liquid chromatography coupled with mass spectrometry. Due to the high selectivity and sensitivity, mass spectrometry coupled to separation techniques such as gas chromatography and liquid chromatography represents a valuable technique for the qualitative and quantitative analysis of chemical substances present in essential oils and plant extracts.

The determination of the chemical composition belonging to essential oils for the following 14 species of the Lamiaceae family of plants: *Hyptis pectinata* [357], *Lavandula angustifolia* [349], *Lavandula officinalis* [356], *Leonurus cardiaca* [290], *Lamium purpureum* [357], *Melissa officinalis* [358], *Mentha spicata* [359], *Marrubium vulgare* [360], *Origanum vulgare* [361], *Ocimum basilicum* [362], *Rosmarinus officinalis* [363], *Salvia officinalis* [364], *Satureja hortensis*, and *Thymus vulgaris* [365], included in most of the scientific articles follow the same steps: (i) collection of flowering aerial parts and drying of the plant material, (ii) hydrodistillation of the dried plant material using a Clevenger apparatus for 1 to 5 hours, (iii) drying the essential oil using anhydrous sodium sulfate (Na_2_SO_4_), storing the essential oil in the dark at 4°C, i.v. injection of 0.1–1 *μ*l of the essential oil in the capillary column of a gas chromatograph, and separation of the chemical compounds, (v) ionization and detection of each volatile substance in a mass spectrometer, and (vi) identification of the components performed based on their retention indices established in relation with a series of *n*-alkanes (C_8_–C_32_) and based on the mass spectra stored in NIST 21, NIST 107, Wiley spectral libraries, or reported in scientific articles.

The volatile substances isolated from the 14 species of plants and analyzed by gas chromatography coupled with mass spectrometry are presented for each of the essential oils in Table 1.

The chemical compounds identified by LC-ESI-MS in extracts prepared for the 9 species of plants that are included in the Lamiaceae family are also presented in Table 2.

Yalçin and the collaborators showed, using HPLC-ESI-MS, that the *n*-butanol extract of *Lamium garganicum* subsp. *Laevigatum*, which was previously shown to possess anti-inflammatory and antinociceptive activity, contains nine iridoid glycosides [366].

The decoction prepared from *Melissa officinalis* dry leaves was filtered through a Whatman no. 4 filter paper, frozen and lyophilized. The phenolic compounds were separated and analyzed by HPLC coupled with an ESI-triple quadrupole-ion trap mass spectrometer using the negative-ion mode. The identification of the phenolic compounds was carried out based on the comparison of their retention time, UV-Vis, and mass spectra with those obtained from solutions prepared with standard substances. For the compounds for which no standard substance was available, the identification was performed based on the scientific literature [367].

Based on the UHPLC-ESI-MS data reported by Martina Cirlini et al. [368], the methanolic extract of *Mentha spicata* contains 88% of rosmarinic acid derivatives when calculating the amount of rosmarinic acid derivatives as percentage of the total amount of detected phenols. For the salvianolic acids, a percentage of 5.6% of the total amount of detected phenols was calculated.

Taamalli and collaborators reported the analyses of the methanolic extract of *Mentha pulegium* performed using an UPLC-ESI-QTOF mass spectrometer coupled with a liquid chromatograph and detected metabolites from the following groups: hydroxybenzoic acids, hydroxycinnamic acids, flavanols, flavones, flavanones, flavonols, organic acids, nucleosides, amino acids, and fatty acids [56]. In the methanolic extract of *Mentha pulegium*, the authors identified a very high amount of gallocatechin.

In the case of the plant *Marrubium vulgare*, Amessis-Ouchemoukh Nadia and collaborators prepared the methanolic extract and analyzed it using an UHPLC-ESI-QTOF instrument. The mass spectra were acquired in the negative-ion mode and showed the presence of the metabolites presented in Table 2 [369].

Anna Vallverdú-Queralt et al. identified the phenolic compounds present in the ethanolic acidified extract of *Origanum vulgare* (Table 2) [370]. After the first extraction with a hydroalcoholic solvent, the extracted plant material was centrifuged, dried, ground, and stored. An aliquot of 1 g of extracted and dried plant material was subjected to extraction, 3 times, with 5 mL of 50% aqueous ethanol containing 0.1% formic acid. All the supernatants were combined, and the organic solvent was evaporated under nitrogen flow. The dried residue was dissolved in 0.1% formic acid and subjected to solid-phase extraction using mixed-mode anion-exchange cartridges in order to reduce potential interferences from plant extracts. For accurate mass measurement, the separation and mass spectrometric analyses were performed using a LC-ESI-LTQ-Orbitrap mass spectrometer operated in negative-ion mode. The quantification of the compounds identified was performed using a triple-quadrupole mass spectrometer.

Pandey and Kumar performed extraction of dried leaves of *Ocimum basilicum* using 80% aqueous methanol [371]. A liquid chromatograph coupled to an ESI-Q-TOF mass spectrometer was used for the identification of the compounds, and the results are summarized in Table 2.

## 5. *In Vivo* Evaluation of Phytochemicals Analgesic Activity

Over the decades, just a few studies tried to find alternatives to the classical treatment of pain, such as the application of the Lamiaceae phytochemicals.

*Marrubiin*, the broadly known diterpenoid lactone, has been associated with the bitter principle of the horehound (*Marrubium vulgare*, *M. deserti* de Noe, *M. alysson*, and *M. thessalum*) and other traditionally important Lamiaceae species (*Leonotis leonurus*, *L. nepetifolia*, and *Phlomis bracteosa*) [67, 374–379]. According to recent literature, extensive pharmacological studies have revealed that *marrubiin* shows a suite of activities such as antinociceptive, antispasmodic, antihypertensive, antidiabetic, gastroprotective, anti-inflammatory, antimicrobial, anticancerous, antioxidant, and antihepatotoxic [65, 67, 71–73, 75, 374, 376–378].

Over time, the antinociceptive profile of marrubiin was analyzed in some animal models of pain. De Jesus et al.'s [64] results showed that marrubiin reveals potent and dose-related antinociceptive effects in mice, whose calculated ID50 values (*μ*mol/kg, i.p.) were as follows: 2.2 in the writhing test, 6.6 (first phase) and 6.3 (second phase) in the formalin-induced pain test, and 28.8 when evaluated over the capsaicin test. These findings show that it is more potent than some other well-known analgesic drugs. The antinociception produced by the marrubiin is not reversed by naloxone when analyzed against the writhing test. Its exact mechanism of action remains however still to be determined, but the results suggest that marrubiin, like the hydroalcoholic extract of *M. vulgare*, does not interact with opioid systems.

Analgesic activity success was obtained by reducing lactonic function of the *marrubiin*, in the formation of marrubiinic acid and two esterified derivatives, which have shown significant analgesic effect on the writhing test in mice [68, 374]. The pharmacological studies specified that marrubiinic acid presents an important (*p* < 0.05) and dose-dependent antinociceptive effect, against the writhing test, in intraperitoneal administration, with ID50 value of 12 *μ*mol/kg, being about 11-fold more active than the standard drugs used as reference, but less active than marrubiin [64].

Marrubiinic acid, given orally, at a dose of 50 mg/kg, produced a marked analgesic effect, reducing 76 ± 0.9% of the number of abdominal constrictions induced by acetic acid, which may recommend that it can be well absorbed by the gastrointestinal tract. However, it was not effective in abolishing pain in a nonopioid way, showing the lack of antinociceptive effects in the hot-plate test [64]. When verified against the capsaicin test, it provided more direct evidence of the analgesic potential on neurogenic pain, causing an inhibition of 37.3 ± 3.8% at 10 mg/kg of capsaicin-induced licking, signifying its involvement with the antagonism of vanilloid receptor [74].

The specific mechanism underlying the antinociceptive action of marrubiinic acid has yet to be determined, but it is unlikely that it is associated with the interaction of opioid peptides. Although marrubiinic acid displayed lesser analgesic properties than marrubiin, it is more potent than some clinically used drugs. In summary, these results show that it could be used as a model to obtain new and more potent analgesic drugs [67].

In 2013, the analgesic activity of the aqueous extracts obtained from leaves (AEL) and stems (AES) of *Rosmarinus officinalis*, as well as its isolated compound—rosmarinic acid (RA)—were analyzed by Lucarini et al. [379]. The analysis is based upon abdominal constriction and formalin tests in mice. The extracts were used at doses of 100, 200, and 400 mg·kg^−1^, and the compounds were tested at 10, 20, and 40 mg·kg^−1^. Orally administered AEL, AES, and RA were not significantly active at any of the doses tested during the abdominal constriction test; the acetyl ester derivative of RA presented significant analgesic activity. These data recommend that the analgesic effects of the acetyl derivative of RA function through a peripheral-mediated mechanism. The acetyl ester derivative of RA is theoretically applicable as a new lead compound for the management of pain.

Takaki et al. [23] investigated the antinociceptive effects of rosemary essential oil (REO) using the acetic acid-induced writhing and hot-plate tests in mice. REO is very common in folk medicine because of its antispasmodic, analgesic, antirheumatic, and carminative effects. In the hot-plate test, the administration of REO in doses of 125, 250, and 500 mg/kg revealed unremarkable effects on response latency, whereas control injection of meperidine induced significant antinociceptive effects.

Moreover, the REO inhibited licking and shaking induced by formalin injections. Instead, at doses of 70, 125, and 250 mg/kg, REO displayed a substantial antinociceptive effect in the acetic acid-induced abdominal writhing test compared with control animals. The results suggest that REO possesses peripheral antinociceptive activity. Similarly, Martinez et al. [363] described the antinociceptive effect of this essential oil using a rat model of arthritic pain. The essential oil with intraperitoneal administration in doses of 100, 300, and 600 mg/kg determined a dose-dependent antinociceptive effect, manifested as a remarkable reduction of the dysfunction in the pain-induced functional impairment model in the rat, mostly at high doses. Emami et al. [34] indicate that rosemary essential oil can inhibit carrageenan-induced paw edema tests in rats and acetic acid-induced writhing model of visceral pain and hot-plate tests in mice, suggesting that rosemary essential oil possesses anti-inflammatory and peripheral antinociceptive activity [23, 380, 381].

Investigations of the effects of carnosol as one of the constituents of rosemary essential oil extract have also shown that carnosol inhibited LPS-stimulated nitric oxide production (LPS (lipopolysaccharide)) in Raw 264.7 cells and reduced inflammation [382]. Moreover, carnosol inhibited proinflammatory leukotrienes in intact polymorph nuclear leukocytes [383], inhibited 5-lipoxygenase, antagonized mobilization of intracellular calcium ions, and inhibited cyclooxygenase type 2 (COX2) in inflamed skin in male Balb/C mice [384].

A recent work demonstrated that extracts from *R. officinalis* can control pain by inhibiting its progression during a persistent noxious condition. As an essential characteristic, rosemary extract prevents damage to the nervous system. Thus, rosemary applies effects on the origins of neuropathic pain and offers a mean to directly modulate nervous signaling. The antineuropathic effects are mainly due to the terpenoid fraction in a mecamylamine-reversed manner, suggesting a pharmacodynamic role of nicotinic acetylcholine receptors [385, 386].

Husseini et al. [355] analyzed the effects of *L. officinalis* hydroalcoholic extract on pain induced by formalin and also cyclooxygenase (COX) type 1 and 2 activity in mice. The administration of the extract intraperitoneally in doses of 100, 200, 250, 300, 400, and 800 mg/kg, respectively, produces significant analgesic and anti-inflammatory activity in the chronic phase of the formalin test and also in hot-plate test in mice with no noted effect on the acute phase of the formalin test.

Moreover, this inhibitory effect is equal to the effects of morphine (10 mg/kg, s.c.), dexamethasone (10 mg/kg, i.p.), and indomethacin (10 mg/kg, i.p.). The extract in doses of 100, 200, and 300 mg/kg significantly reduced heat-induced pain and also reduced COX activity in a dose-dependent manner, where the inhibitory effect on COX1 activity was 33% and on COX2 activity was 45%. Therefore, these results indicate that the possible mechanism of analgesic and anti-inflammatory effects of the extract may be through modulation of COX2 activity.

Other studies [349] have also revealed that the extract of *L. officinalis* leaves might inhibit the formalin-induced chronic pain, abdomen writhing, and carrageenan-evoked edema. High doses of the essential oils and polyphenolic fraction of *L. officinalis* have similar effects by blocking acetic acid evoked pain [353]. This pharmacological activity could be derived from the contribution of various active principles composing the whole oil such as linalool, myrcene, and 1–8 cineole, previously proved to possess antinociceptive proprieties [387–389]. However, administration of the essential oil with naloxone, atropine, and mecamylamine could eliminate the analgesic effect of the extract, which indicates that the analgesic activity of the extract is dependent on cholinergic and opioid systems [349].

The antinociceptive and analgesic effects of the essential oil of *Mentha* spp. (EOM) leaves and its major constituent, piperitenone oxide (PO), were investigated in mice [390]. After an oral administration of 200 mg/kg of EOM and PO, the antinociceptive activity was demonstrated by an important reduction in the acetic acid-induced number of writhings and the second phase of the formalin test, while in the similar range of doses, they did not interfere with the nociception associated with the hot-plate and tail immersion tests. The hot-plate and tail immersion tests are reported to be useful tests in discriminating analgesic agents acting primarily at the spinal medulla level and at higher central nervous system levels (positive results) from those acting through peripheral mechanisms (negative results) [391].

These findings suggest that EOM and PO are acting by peripheral mechanisms. In addition, EOM caused a reduction in the paw licking time for the second phase of the formalin test, when administered at higher doses (100 and 200 mg/kg). At 100 and 200 mg/kg, PO reduced this second phase to 8.3 ± 2.7 s (*N* = 12) and 3.0 ± 1.2 s (*N* = 10), respectively. The antinociceptive activity induced by EOM and PO in the writhing and formalin tests was not altered by naloxone, demonstrating that their actions do not depend on opioid receptors [392], supporting the anti-inflammatory hypothesis for their mechanism of action. Thus, it is reasonable to suggest that EOM and PO have an analgesic activity, which is probably indirect and attributed to the anti-inflammatory activity, which does not involve the central nervous system [393].

## 6. Future Perspectives and Conclusions

The Lamiaceae family includes numerous known species that are used as traditional medicine. The present review summarizes the general aspects, traditional uses, pharmacology, and *in vitro* and *in vivo* studies of *Betonica officinalis, Glechoma hederacea, Hyptis pectinata, Lavandula* genus*, Leonurus cardiaca, Lamium* genus*, Melissa officinalis, Mentha* genus*, Marrubium vulgare, Origanum* genus*, Ocimum* genus*, Rosmarinus officinalis, Salvia* genus*, Satureja hortensis, Stachys lavandulifolia, Scutellaria lateriflora, Sideritis* genus*, Teucrium* genus*, Thymus* genus, and *Ziziphora tenuior*, belonging to Lamiaceae botanical genus. The above-referred studies reported that the abovementioned medicinal plants have potent analgesic and antinociceptive activity. The findings of this review are promising, regarding new potential therapeutic agents with possible modulation in pain therapy. Most of the extracts identified did not present any toxic capabilities or known side effects and were at least as efficient as currently used synthetic drugs. Overall, although promising information evidence the efficacy of Lamiaceae genus in the treatment of pain associated disorders, the data are too preliminary and mostly fail to explain the exact cellular and molecular mechanisms of action and the respective active compounds. Therefore, future studies should be focused on investigating mechanisms of actions, realistic dosages, clinical efficacy, and safety of the extracts and active compounds in pain treatment. This review covers a useful approach for further identification of new compounds from various medicinal plants, which may be effective in pain management.

## Figures and Tables

**Table 1:** Compounds identified by GC-MS in essential oil.

Number	Compound	Plant name														
		*Hyptis pectinata*	*Lavandula angustifolia*	*Lavandula officinalis*	*Leonurus cardiaca*	*Lamium purpureum*	*Melissa officinalis*	*Mentha spicata*	*Marrubium vulgare*	*Origanum vulgare*	*Ocimum basilicum*	*Rosmarinus officinalis*	*Salvia officinalis*	*Satureja hortensis*	*Thymus vulgaris*	References
1	Sabinene	+				+	+	+		+			+			[355, 357–359, 361, 364]
2	*β*-Pinene	+	+	+	+	+	+	+				+	+	+		[355, 357, 359–361, 363–365]
3	1-Octen-3-ol	+					+			+						[355, 358, 361]
4	Limonene	+			+	+	+	+					+	+	+	[290, 355, 357, 358, 364, 365]
5	*β*-(E)-Ocimene	+	+			+	+			+						[349, 355, 357, 358, 361]
6	Linalool	+	+	+					+	+	+	+	+		+	[349, 355, 356, 360–363]
7	*α*-Copaene	+		+		+	+				+					[355, 356, 357, 358, 362]
8	*β*-Elemene	+				+					+					[355, 357, 362]
9	*β*-Caryophyllene	+	+	+	+	+	+	+	+	+		+	+	+	+	[349, 355–359, 361, 363–365]
10	Aromadendrene	+											+			[355, 364]
11	cis-Muurola-3,5-diene	+														[355]
12	*α*-Humulene	+		+	+		+		+	+	+		+			[290, 355, 356, 358, 360–362, 364]
13	Germacrene D	+	+		+	+	+		+	+	+					[290, 349, 355, 358, 360–362]
14	cis-*β*-Guaiene	+														[355]
15	*γ*-Patchoulene	+														[355]
16	Germacrene A	+														[355]
17	*γ*-Cadinene	+			+	+	+			+	+					[290, 355, 357, 358, 361, 362]
18	trans-Calamenene	+														[355]
19	Germacrene B	+		+												[355, 356]
20	Caryophyllene oxide	+			+		+						+			[290, 355, 358, 364]
21	1,10-Di-epi-cubenol	+														[355]
22	Calamusenone	+														[355]
23	Cyperotundone	+														[355]
24	*α*-Thujene		+			+				+		+	+	+		[349, 357, 361, 363–365]
25	*α*-Pinene		+	+	+	+	+	+	+	+		+	+	+	+	[290, 349, 356–361, 363–365]
26	*α*-Fenchene		+										+			[349, 364]
27	Camphene		+	+					+	+		+	+	+	+	[349, 356, 360, 361, 363–365]
28	Delta-3-carene		+													[349]
29	1,8-Cineole		+	+			+	+	+		+	+	+			[349, 356, 358–360, 362–364]
30	*γ*-Terpinene		+	+			+	+		+			+	+	+	[349, 356, 358, 359, 361, 364, 365]
31	Terpinolene		+		+		+					+		+	+	[290, 349, 358, 363–365]
32	Camphor		+	+					+			+	+			[349, 356, 360, 363, 364]
33	Borneol		+						+			+	+	+	+	[349, 360, 363–365]
34	Lavandulol		+													[349]
35	4-Terpineol		+	+			+	+					+	+		[349, 356, 358, 359, 364, 365]
36	*α*-Terpineol		+	+			+	+		+			+	+	+	[349, 356, 358, 359, 361, 364, 365]
37	Linalyl acetate		+							+			+			[349, 361, 364]
38	Bornyl acetate		+	+			+				+	+	+			[349, 356, 358, 362–364]
39	cis-*β*-Farnesene		+													[349]
40	Verbenene			+												[356]
41	1,3,5-cycloheptatriene			+												[356]
42	3-Octanone			+		+										[356, 357]
43	*β*-myrcene			+		+	+	+		+		+	+	+	+	[356, 357–359, 361, 364–365]
44	3-Octanol			+		+										[356, 357]
45	*α*-Phellandrene			+						+		+		+		[356, 361, 363, 365]
46	*o*-Isopropenyl toluene			+												[356]
47	*α*-Terpinene			+			+			+				+	+	[356, 358, 361, 364, 365]
48	*p*-Cymene			+	+		+	+		+			+	+	+	[290, 356, 358, 359, 361, 364, 365]
49	Linalool oxide			+												[356]
50	Methyl benzoate			+												[356]
51	Thujancis			+												[356]
52	D-fenchyl alcohol			+												[356]
53	Pinocarveol			+												[356]
54	Isopinocamphone			+									+			[356, 364]
55	Pinocarvone			+												[356]
56	Pinocamphone			+									+			[356, 364]
57	Naphthalene			+												[356]
58	*p*-Cymen-8-ol			+									+			[356, 364]
59	Myrtenol			+												[356]
60	Verbenone			+												[356]
61	*trans*-Carveol			+				+								[356, 359]
62	*β*-Citronellol			+					+							[356, 360]
63	Pulegone			+			+	+								[356, 358, 359]
64	Piperitone			+				+								[356, 359]
65	Cinnamaldehyde			+												[356]
66	Thymol			+			+			+				+	+	[356, 358, 361, 364, 365]
67	2-Hydroxy-4-isopropyl-1-methylbenzene			+												[356]
68	Carvacrol			+						+			+	+	+	[356, 361, 364, 365]
69	Piperitenone			+			+									[356, 358]
70	*α*-Cubebene			+												[356]
71	Thymyl acetate			+												[356]
72	Farnesene			+		+					+					[356, 357, 362]
73	*β*-Acoradiene			+												[356]
74	*α*-Cedrene			+												[356]
75	Spathulenol			+	+		+				+			+		[290, 356, 358, 362, 365]
76	Butylidenephthalide			+												[356]
77	3N Butylphthalide			+												[356]
78	Butylidene dihydrophthalide			+												[356]
79	3-Methyl butanal				+											[290]
80	(E,E)-2,4-Hexadienal				+											[290]
81	Benzaldehyde				+											[290]
82	Dehydro-1,8-cineole				+											[290]
83	Phenylacetaldehyde				+											[290]
84	*p*-Menth-1,5-dien-8-ol				+											[290]
85	Dodecane				+											[290]
86	Dihydroedulan I				+											[290]
87	(E)-*α*-ionone				+											[290]
88	*β*-Humulene				+											[290]
89	Geranyl acetone				+											[290]
90	(E)-*β*-ionone				+											[290]
91	*β*-Selinene				+											[290]
92	Eugenyl acetate				+											[290]
93	(E)-nerolidol				+											[290]
94	*trans*-Sesquisabinene hydrate				+											[290]
95	Presilphiperfolan-8-ol				+											[290]
96	*β*-Atlantol				+											[290]
97	Epi-cedrol				+											[290]
98	*α*-Bisabolol				+											[290]
99	(2E, 6E)-farnesol				+											[290]
100	8-Cedren-13-ol acetate				+											[290]
101	6,10,14-Trimethyl-2-pentadecanone				+											[290]
102	(2Z, 6E)-farnesyl acetate				+											[290]
103	Methyl-linolenate				+											[290]
104	Allo-aromadendrene epoxide				+											[290]
105	Eicosane				+											[290]
106	Heneicosane				+											[290]
107	(E)-3-hexen-1-ol					+										[349]
108	Heptanal					+										[357]
109	1-Octen-3-one					+										[357]
110	(E,E)-2,4-Heptadienal					+										[357]
111	Z-*β*-ocimene					+	+			+						[356, 357, 361]
112	Undecane					+										[357]
113	Nonanal					+										[357]
114	Decanal					+										[357]
115	*β*-Cyclocitral					+										[357]
116	N-tridecane					+										[357]
117	Delta-elemene					+				+						[357, 361]
118	(E)-*β*-damascenone					+										[357]
119	*β*-bourbonene					+		+	+	+						[290, 357, 359, 361]
120	(E)-*β*-farnesene					+										[357]
121	Farnesane					+										[357]
122	Bicyclogermacrene					+	+							+		[357, 358, 365]
123	*n*-Pentadecane					+										[357]
124	(E)-hydrate de sabinene						+									[358]
125	*p*-Menth-3,8-diene						+									[358]
126	Z-hydrate de sabinene						+						+			[358, 365]
127	1-Octen-3-ol acetate						+									[358]
128	3-Octenyl acetate						+									[358]
129	*p*-Menth-3-en-8-ol						+									[358]
130	Menthone						+	+								[358, 359]
131	Isomenthone						+	+	+						+	[358–360, 364]
132	Neomenthol						+									[358]
133	Z-Piperitone oxide						+									[358]
134	Pseudodisphenol						+									[358]
135	Isopulegyl acetate 1						+									[358]
136	Neomenthylacetate						+	+								[358, 359]
137	Diosphenol						+									[358]
138	Menthylacetate						+	+								[358, 359]
139	Piperitenone oxide						+									[358]
140	(E)-jasmone						+									[358]
141	Nepetalactone						+									[358]
142	*p*-Menth-1,2,3-triol						+									[358]
143	*α*-Gurjunene						+									[358]
144	Cadina-3,5-diene						+									[358]
145	Delta-cadinene						+		+							[358, 360]
146	Viridiflorol						+									[358]
147	1,10-Di-epi-cubenol						+									[358]
148	*α*-cadinol						+				+					[358, 362]
149	2-Octanol							+								[359]
150	Menthol							+								[359]
151	Isomenthol							+								[359]
152	Dihydrocarveol							+					+		+	[359, 364]
153	Carvone							+		+						[357, 359]
154	*cis*-Carveol							+								[359]
155	*trans*-Anethole							+								[359]
156	Menthyl camphor							+								[359]
157	Eugenol							+			+					[359, 362]
158	*N*-trimethylsilyl trifluoroacetamide								+							[360]
159	*N*,*Nʹ*-bis(trimethylsilyl) trifluoroacetamide								+							[360]
160	*α*-Thujone								+							[360]
161	1-Vinyl cyclohexane								+							[360]
162	Geraniol								+				+			[360, 364]
163	Citronellyl formate								+							[360]
164	*α*-Muurolene								+							[360]
165	*α*-Amorphene								+		+					[360, 362]
166	Neoalloocimene								+							[360]
167	Neryl acetate								+							[360]
168	Ledene								+							[360]
169	*β*-Bisabolene								+	+				+		[360, 361, 365]
170	*α*-Agarofuran								+							[360]
171	*γ*-Eudesmol								+							[360]
172	Furan-2-one, 4-phenyltetrahydro								+							[360]
173	*β*-Cubebene								+							[360]
174	Citronellyl butanoate								+							[360]
175	Geranyl tiglate								+							[360]
176	Cyclononasiloxane, octadecamethyl								+							[360]
177	Eicosamethylcyclodecasiloxane								+							[360]
178	*β*-Phellandrene									+			+		+	[361, 365]
179	Carvacrol methyl ether									+				+		[361, 365]
180	Allo-aromadendrene									+	+		+			[361, 362, 364]
181	*cis*-Epoxy-ocimen										+					[362]
182	*cis*-Caryophyllene										+					[362]
183	*α*-Guaiene										+					[362]
184	Delta-guaiene										+					[362]
185	*cis*-Calamenene										+					[362]
186	Epi-bicyclosesquiphellandrene										+					[365]
187	*β*-Eudesmol										+					[362]
188	*α*-Selinene										+					[362]
189	Zingiberene										+					[362]
190	Tricyclene											+				[360]
191	*o*-Cymene											+	+		+	[358, 363, 364]
192	Trifluoroacetyl-α-															
Terpineol											+					[363]
193	Carene											+				[363]
194	*cis*-*α*-Terpineol											+				[363]
195	2-Bornanone											+				[363]
196	Isoborneol											+			+	[363, 364]
197	*trans*-Pinocarveol												+			[364]
198	(-)-Citronellal												+		+	[363]
199	Myrtenal												+		+	[363]
200	Myrtenol												+		+	[363]
201	Isobornyl acetate												+			[363]
202	*β*-Cedrene												+			[363]
203	*γ*-Gurjunene												+			[363]
204	*α*-7-Epi-selinene												+			[363]
205	Carvacrol acetate													+		[361]

**Table 2:** Compounds identified by HPLC-ESI-MS in aqueous and alcoholic extracts.

Number	Compound	Plant name									
		*Lamium garganicum* subsp. *Laevigatum*	*Melissa officinalis*	*Mentha spicata*	*Mentha pulegium*	*Marrubium vulgare*	*Origanum vulgare*	*Ocimum basilicum*	*Rosmarinus officinalis*	*Salvia officinalis*	References
1	Lamalbidee	+									[366]
2	Sesamoside	+									[366]
3	Lamiide	+									[366]
4	6-*β*-OH ipolamiide	+									[366]
5	Shanzhiside methyl ester	+									[366]
6	Dehydropenstemoside	+									[366]
7	8-*O*-acetyl shanzhiside methyl ester	+									[366]
8	6-Syringyl-8-*O*-acetyl shanzhiside methyl ester	+									[366]
9	3-(3,4-Dihydroxyphenyl)-lactic acid		+								[367]
10	Caftaric acid		+	+							[367, 372]
11	Caffeic acid hexoside		+				+				[367, 370]
12	Fertaric acid		+								[367]
13	Caffeic acid		+	+		+	+	+	+	+	[367, 369, 370, 372, 373]
14	Sulfated rosmarinic acid		+								[367]
15	Yunnaneic acid E		+								[367]
16	Prolithospermic acid		+								[367]
17	Lithospermic acid A isomer		+								[367]
18	Chicoric acid		+								[367]
19	Salvianolic acid C derivative		+								[367]
20	Sagerinic acid		+	+							[367, 372]
21	*cis*-Rosmarinic acid		+								[367]
22	*trans*-Rosmarinic acid		+								[367]
23	Salvianolic acid A isomer		+								[367]
24	Protocatechuic acid			+			+	+		+	[370–373]
25	Hydroxybenzoic acid			+						+	[372, 373]
26	Hydroxyphenyllactic acid			+							[372]
27	Luteolin-8-C-glucoside (orientin)			+							[372]
28	3′-Caffeoylquinic (neochlorogenic acid)			+			+				[370, 372]
29	Coumaric acid			+			+				[370, 372]
30	Salvianolic acid F			+							[372]
31	Dicaffeic acid			+							[372]
32	5′-Caffeoylquinic (chlorogenic acid)			+	+		+	+		+	[56, 370–373]
33	Feruloylquinic acid			+							[372]
34	Rosmarinic acid-*O*-caffeic acid			+							[372]
35	Rosmarinic acid-rutinoside			+							[372]
36	Salvianolic acid J isomer			+							[372]
37	Luteolin-rutinoside			+	+				+		[56, 372]
38	Rosmarinic acid-*O*-hexoside			+			+				[370, 372]
39	Luteolin-hexoside			+					+	+	[372, 373]
40	Luteolin-7-glucuronide			+				+	+	+	[371–373]
41	Salvianolic acid B/E isomer			+							[372]
42	Narirutin (naringenin-7-*O*-rutinoside)			+							[372]
43	Salvianolic acid D			+							[372]
44	Salvianolic acid E			+	+						[56, 372]
45	Rosmarinic acid			+	+		+	+	+	+	[56, 370–373]
46	Sagerinic acid isomer			+							[372]
47	Salvianolic acid A derivative			+							[372]
48	Lithospermic acid			+	+						[56, 372]
49	Salvianolic acid B			+	+						[56, 372]
50	Dehydrorosmarinic acid			+							[372]
51	Rosmarinic acid dihexoside			+							[372]
52	Salvianolic acid A			+							[372]
53	Apigenin				+	+	+	+	+		[56, 369–372]
54	Hydroxybenzoic acid hexose isomer 1				+					+	[56, 373]
55	Vanillyl alcohol				+						[8]
56	Dihydroxybenzoic acid hexose				+						[8]
57	Vanillic acid hexose				+		+				[4, 8]
58	Syringic acid				+		+	+			[56, 370, 371]
59	Hydroxybenzoic acid hexose isomer 2				+						[8]
60	Dihydroxybenzoic acid				+						[8]
61	Syringic acid hexose				+						[8]
62	Caffeic acid glucuronide isomer 1				+						[8]
63	Caffeic acid glucuronide isomer 2				+						[56]
64	*p*-Coumaric acid				+			+	+		[56, 371, 372]
65	Salvianolic acid I				+						[56]
66	Salvianolic acid H				+						[56]
67	Isosalvianolic acid B				+						[56]
68	Eukovoside				+						[56]
69	Salvianolic acid C				+						[56]
70	Catechin-4-ol-*O*-glycopyranoside				+						[56]
71	Gallocatechin isomer 1				+						[56]
72	(+)-Catechin hydrated				+						[56]
73	Diosmin				+						[56]
74	Acacetin rutinoside				+						[56]
75	Hesperidin				+						[56]
76	Isosakuranetin-*O*-rutinoside				+						[56]
77	Syringetin				+						[56]
78	Jaceidin isomer 1				+						[56]
79	Geshoidin					+					[369]
80	Decaffeoylverbascoside					+					[369]
81	Sacranoside A					+					[369]
82	Marruboside					+					[369]
83	Forsythoside B (isomer 1)					+					[369]
84	Forsythoside B (isomer 2)					+					[369]
85	Verbascosin					+					[369]
86	Luteolin-*O*-glucoside					+				+	[369, 373]
87	Alyssonoside					+					[369]
88	Leukoceptoside A					+					[369]
89	Apigenin-*O*-glucoside					+					[369]
90	Deacetylforskolin (isomer 1)					+					[369]
91	Preleosibirin					+					[369]
92	Deacetylforskolin (isomer 2)					+					[369]
93	Garcinone E (isomer 1)					+					[369]
94	Terniflorin (isomer 1)					+					[369]
95	Garcinone E (isomer 2)					+					[369]
96	Luteolin					+		+	+		[369, 371, 372]
97	Premarrubiin (isomer 1)					+					[369]
98	Terniflorin (isomer 2)					+					[369]
99	Premarrubiin (isomer 2)					+					[369]
100	Deacetylforskolin (isomer 3)					+					[369]
101	Terniflorin (isomer 3)					+					[369]
102	Deacetylforskolin (isomer 4)					+					[369]
103	Deacetylforskolin (isomer 5)					+					[369]
104	Marrulibacetal A (isomer 1)					+					[369]
105	Marrulibacetal A (isomer 2)					+					[369]
106	Premarrubiin (isomer 3)					+					[369]
107	Premarrubiin (isomer 4)					+					[369]
108	Anisofolin A (isomer 1)					+					[369]
109	Anisofolin A (isomer 2)					+					[369]
110	Marrubenol					+					[369]
111	Gallic acid						+	+			[372, 373]
112	*p*-Hydroxybenzoic acid						+	+	+		[371, 372]
113	*m*-Hydroxybenzoic acid						+				[370]
114	Coumaric acid-*O*-hexoside						+				[370]
115	Cryptochlorogenic acid (4-*O*-caffeoylquinic acid)						+				[370]
116	Homovanilic acid						+				[370]
117	Apigenin-C-hexoside-C-hexoside						+				[370]
118	4-*O*-*p*-coumaroylqunic acid						+				[370]
119	Kaempferol-*O*-dihexoside						+				[370]
120	Ferulic acid						+	+			[370, 371]
121	Kaempferol-3-*O*-rutinoside						+	+			[370, 371]
122	Quercetin-3-*O*-glucoside						+	+			[370, 371]
123	Kaempferol-3-*O*-glucoside						+				[370]
124	Dicaffeoylquinic acid						+				[370]
125	Hesperidin						+				[370]
126	Apigenin-7-*O*-glucoside						+	+	+		[370–372]
127	Kaempferol						+	+			[370, 371]
128	Quercetin						+	+			[370, 371]
129	Hesperidin (hesperetin-7-*O*-rutinoside)						+		+		[370, 371]
130	Rosmanol						+		+		[370, 372]
131	Carnosic acid						+		+	+	[370, 372, 373]
132	Quercetin-3-*O*-rutinoside (rutin)							+			[371]
133	Quercetin-3-*O*-malonylglucoside							+			[371]
134	Apigenin-7-*O*-glucuronide							+		+	[371, 373]
135	Eugenol							+			[371]
136	Coniferaldehyde							+			[371]
137	Isothymusin							+			[371]
138	Kaempferide							+			[371]
139	Cirsiliol							+			[371]
140	Cirsimaritin							+			[371]
141	Cirsilineol							+			[371]
142	Nevadensin							+			[371]
143	Acacetin							+			[371]
144	5-Desmethylsinensetin							+			[371]
145	Methyleugenol							+			[371]
146	Salvigenin							+			[371]
147	Methoxyeugenol							+			[371]
148	Gardenin B							+			[371]
149	Apigenin-7,4-dimethylether							+			[371]
150	Oleanolic acid							+			[371]
151	Ursolic acid							+			[371]
152	Galloylglucose							+			[371]
153	Etylprotocatechuate							+			[371]
154	Methylgallate							+			[371]
155	Sinapinic acid							+			[371]
156	Methylprotocatechuate							+			[371]
157	Vanillic acid							+			[371]
158	Ethyl caffeate							+			[371]
159	Medioresinol								+		[372]
160	Isorhamnetin-3-*O*-hexoside								+		[372]
161	Homoplantaginin (hispidulin 7-glucoside)								+		[372]
162	Dihydroxy-dimethoxyflavone derivative								+		[372]
163	Dihydroxy-dimethoxyflavone								+		[372]
164	Medioresinol derivative								+		[372]
165	Luteolin-3′-acetyl-*O*-glucuronide								+		[372]
166	Medioresinol-glucuronide								+		[372]
167	Eriodictyol								+		[372]
168	Isorhamnetin-rutinoside								+		[372]
169	Isorhamnetin								+		[372]
170	Trihydroxy-methoxyflavone								+		[372]
171	Methylrosmarinate								+		[372]
172	Apigenin-7-*O*-rutinoside								+		[372]
173	Hispidulin-rutinoside								+		[372]
174	Hesperetin								+		[372]
175	5,6,7,10-Tetrahydro-7-hydroxyrosmariquinone derivative								+		[372]
176	Cirsimaritin								+		[372]
177	Carnosol methyl ether isomer								+		[372]
178	Rosmanol quinone								+		[372]
179	Epirosmanol								+		[372]
180	Carnosol quinone								+		[372]
181	Isosakuranetin								+		[372]
182	Genkwanin								+		[372]
183	Carnosic acid hexoside								+		[372]
184	Rosmanol isomer								+		[372]
185	Carnosol								+		[372]
186	Carnosic acid quinone								+		[372]
187	4′-Methoxytectochrysin								+		[372]
188	Rosmadial								+		[372]
189	Rosmaridiphenol								+		[372]
190	5,6,7,10-Tetrahydro-7-hydroxyrosmariquinone								+		[372]
191	12-*O*-methylcarnosic acid								+		[372]
192	Betulinic acid								+		[372]
193	Salvianic acid A									+	[373]
194	Protocatechuoyl-hexose									+	[373]
195	Dimethoxybenzoic acid									+	[373]
196	Coumaroyl hexose									+	[373]
197	Caffeoyl-fructosyl-glucose									+	[373]
198	Coumaroyl-apiosyl-glucose-isomer									+	[373]
199	Chlorogenic acid isomer									+	[373]
200	Methyldihydrojasmonic acid isomer									+	[373]
201	Feruloyl-glucose isomer									+	[373]
202	Salvianolic acid I isomer									+	[373]
203	Saponarin (apigenin-6-C-glucoside-7-*O*-glucoside)									+	[373]
204	Salvianolic F isomer									+	[373]
205	Luteolin diglucuronide									+	[373]
206	Eriocitrin									+	[373]
207	Hydroxy-luteolin-glucuronide									+	[373]
208	Methylmelitric acid A									+	[373]
209	Apigenin-diglucuronide									+	[373]
210	Sagecoumarin									+	[373]
211	Luteolin-rutinoside isomer									+	[373]
212	Apigenin rutinoside isomer									+	[373]
213	Apigenin hexoside									+	[373]
214	Hispiludin-glucuronide									+	[373]
215	Salvianolic acid B									+	[373]
216	Salvianolic acid K isomer									+	[373]
217	Carnosol isomer									+	[373]
218	Epirosmanol									+	[373]
219	Carnosic acid isomer									+	[373]
